# Association between ambient particulate matter exposure and semen quality in fertile men

**DOI:** 10.1186/s12940-022-00831-5

**Published:** 2022-01-16

**Authors:** Wei Wu, Yiqiu Chen, Yuting Cheng, Qiuqin Tang, Feng Pan, Naijun Tang, Zhiwei Sun, Xinru Wang, Stephanie J. London, Yankai Xia

**Affiliations:** 1 State Key Laboratory of Reproductive Medicine, Institute of Applied Toxicology, School of Public Health, Nanjing Medical University, 101 Longmian Avenue, Nanjing, 211166 China; 2 Key Laboratory of Modern Toxicology of Ministry of Education, School of Public Health, Nanjing Medical University, Nanjing, China; 3 Department of Health and Human Services, National Institute of Environmental Health Sciences, National Institutes of Health, Research Triangle Park, Durham, USA; 4 Department of Obstetrics, The Affiliated Obstetrics and Gynecology Hospital of Nanjing Medical University, Nanjing Maternity and Child Health Care Hospital, Nanjing, China; 5 Department of Urology, The Affiliated Obstetrics and Gynecology Hospital of Nanjing Medical University, Nanjing Maternity and Child Health Care Hospital, Nanjing, China; 6 Department of Occupational and Environmental Health, School of Public Health, Tianjin Medical University, Tianjin, China; 7 Beijing Key Laboratory of Environmental Toxicology, School of Public Health, Capital Medical University, Beijing, China

**Keywords:** Ambient air pollution, PM_2.5_, PM_10_, Fertility, Semen quality, Sperm motility

## Abstract

**Background:**

Several studies have suggested adverse effects of particulate matter (PM) exposure on male reproductive health; few have investigated the association between PM exposure and semen quality in a large population of fertile men.

**Methods:**

We evaluated 14 parameters of semen quality in 1554 fertile men in Nanjing from 2014 to 2016. Individual exposure to particular matter ≤10 μm in diameter (PM_10_) and ≤ 2.5 μm in diameter (PM_2.5_) during key periods of sperm development (0-90, 0-9, 10-14, 15-69, and 70-90 days before semen collection) were estimated by inverse distance weighting interpolation. Associations between PM exposure and semen quality were estimated using multivariable linear regression.

**Results:**

Higher 90-days average PM_2.5_ was in association with decreased sperm motility (2.21% for total motility, 1.93% for progressive motility per 10 μg/m^3^ increase, *P* <  0.001) and four quantitative aspects of sperm motion (curvilinear velocity (VCL), straight line velocity (VSL), average path velocity (VAP), and amplitude of lateral head displacement (ALH), *P* <  0.01). The association between PM_2.5_ exposure and semen quality were generally stronger for the earlier exposure window (70-90 days prior to ejaculation) than for recent exposure (0-9, 10-14, or 15-69 days). In the subgroup of men who had normal sperm parameters (*n* = 1019), similar results were obtained. Ninety-days PM_10_ exposure was associated only with decreased VCL and VAP and was not related to sperm concentration.

**Conclusions:**

Exposure to PM_2.5_ adversely affects semen quality, specifically lower sperm motility, in fertile men.

**Graphical abstract:**

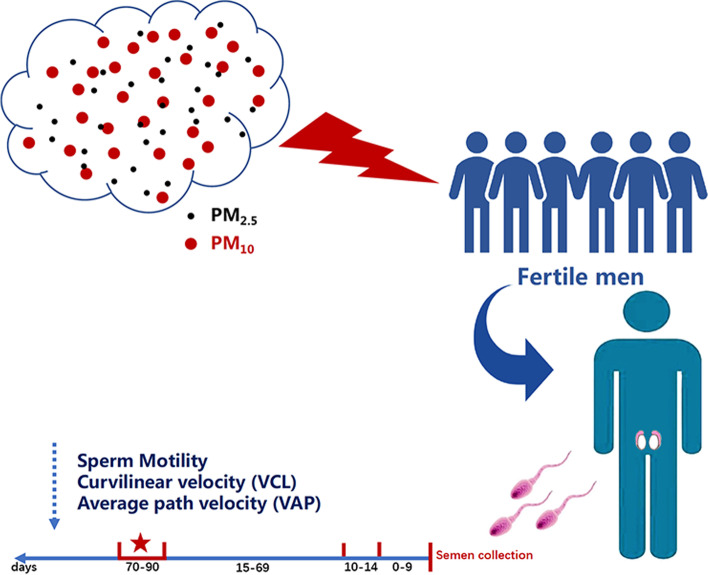

**Supplementary Information:**

The online version contains supplementary material available at 10.1186/s12940-022-00831-5.

## Background

Male factors are responsible for about 50% of infertility in couples and male infertility is an important public health issue worldwide [1]. A significant decline in human semen quality has been observed over the past 70 years, even in fertile men [2, 3]. Exposures that have been associated with reduced semen quality include air pollutants, smoking, and heavy metals [4–6]. The World Health Organization (WHO) reports that 1.4 billion urban residents live in areas with air quality that does not meet WHO air quality guidelines [7]. China has experienced deterioration of the air quality due to its rapid socioeconomic development. The situation is especially notable in the Yangtze River Delta, one of the areas in China undergoing most rapid urbanization [8].

A large body of literature documents the association between ambient air pollution and a range of important health conditions including cardiovascular and respiratory diseases [9–12] and cancers [13]. A growing body of literature suggests that exposure to ambient air pollutants during pregnancy increases the risk of adverse birth outcomes [14, 15]. Sentinel animal studies provide cogent evidence that ambient air pollution exposure can damage male germ cells [16]. Particulate matter (PM) is a key component of air pollution and various diseases are associated with it [17]. Pires et al. showed that fine particulate matter (PM_2.5_) levels in Sao Paulo adversely affects spermatogenesis in mice, but they didn’t investigate the effects of PM_10_ exposure [18]. During the past few years, there has been increasing interest in the effects of air pollution on male reproductive health [19, 20]. In humans, several studies have reported changes in sperm parameters, such as sperm mobility and movement, in relation to exposure to air pollution, providing evidence for exposure-related reductions in sperm quality [4, 21–23]. However, the reported effects of PM exposure on the male reproductive system are inconsistent [19, 24, 25]. A number of studies have focused on men being evaluated for infertility, but studies in men known to be fertile are few. Furthermore, most of these studies focused only on a few semen parameters. These limitations may impair the identification of true associations between PM exposure and semen quality.

Because development of sperm takes approximately 3 months [26], most studies have analyzed PM exposure in the 90 days before semen examination [25, 27, 28]. It is known that the sperm development covers four key stages before semen ejaculation: 0-9 (epididymal storage), 10-14 (development of sperm motility), 15-69 (spermatogenesis stage II), and 70-90 days (spermatogenesis stage I). However, few researchers have considered these different exposure stages within the 90-days window [29–31], and the conclusions are inconsistent.

We therefore investigated the association between ambient PM (PM_2.5_ and PM_10_) exposure, in the 90 days prior to semen collection and semen quality in a large population of men in Nanjing, China known to be fertile and attempted to clarify which stage of sperm development is most impacted by PM exposure. Our large cohort with participants of known fertility would make the results more representative.

## Methods

### Study population

The study population initially consisted of 1607 fertile men from Nanjing Medical University Longitudinal Investigation of Fertility and the Environment (NMU-LIFE) study from January 1st, 2014 to December 31st, 2016. The NMU-LIFE was established in September 2010 to examine the effects of environmental and lifestyle factors on reproductive health and birth outcomes in the offspring. The study area of NMU-LIFE locates in Yangtze River Delta Region, China. Pregnant women that went for registration at the hospital were identified as candidates for the study. Maternity care doctors determined the eligible individuals. Exclusion criteria included maternal age < 20 or > 45 years, non-permanent residents and intention of delivering in other cities. After learning about the study in details, the woman that agreed to participate would represent herself and her family members to sign an informed consent, which meant the whole family was recruited. The information collected in NMU-LIFE include the basic information and disease information of fertile males, pregnant women, and their children, as well as biospecimen including blood, urine, semen, placenta and follicular fluid (more detailed information about the cohort can be found in supplementary material). We excluded 21 men without complete semen reports, 17 with missing examination dates, and 15 without exact addresses, leaving 1554 men for analysis. All of the 1554 ferile men had fathered at least one healthy child within the previous year. All male participants in our present study were without a history of treatment. The study was approved by the Institutional Ethics Committee of Nanjing Medical University. All study participants gave written informed consent. All activities involving human subjects were conducted under full compliance with government policies and the Declaration of Helsinki.

### Data sources

**Fig. 1:**
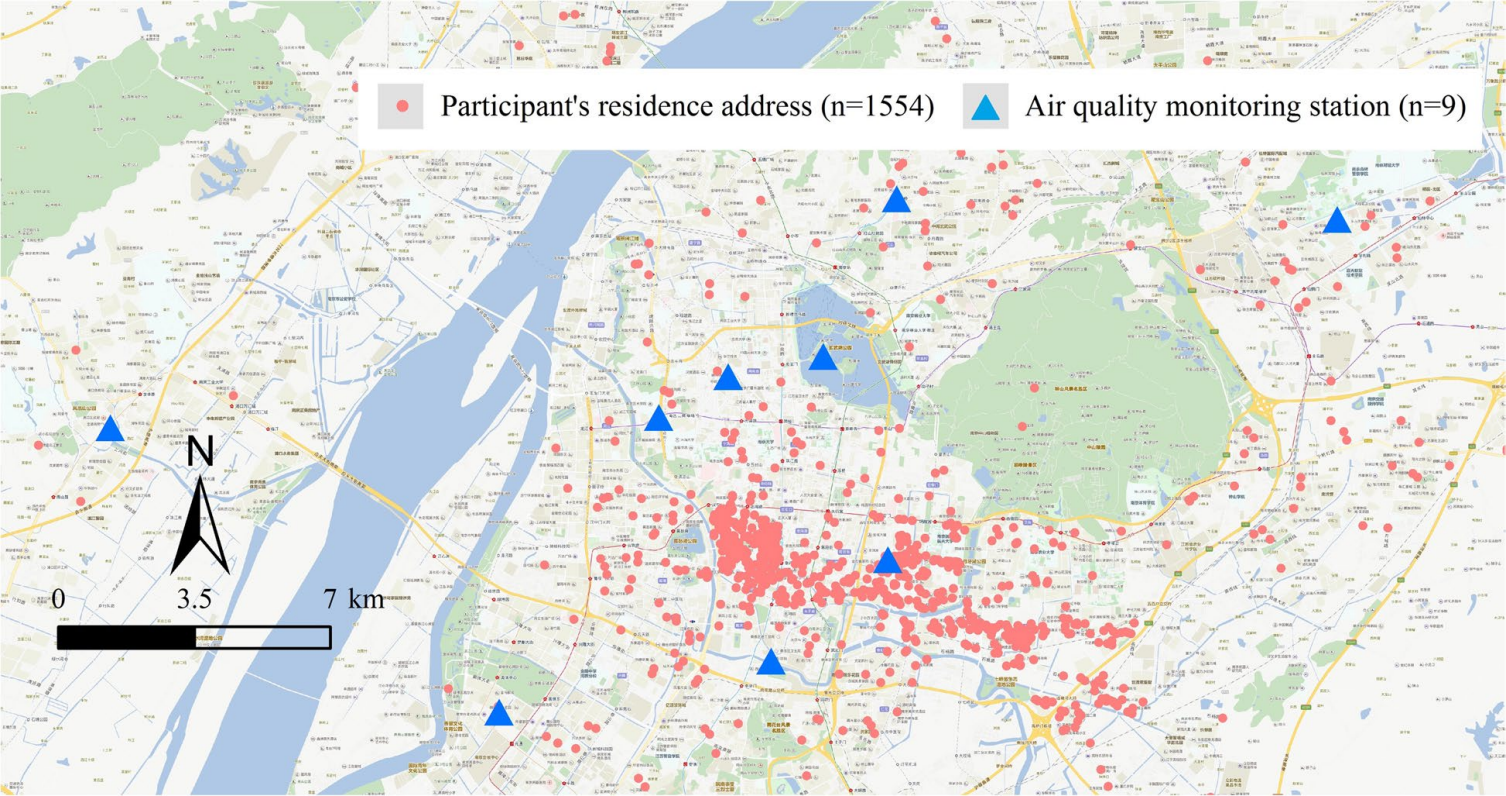
Spatial distribution of nine fixed ambient air quality monitoring stations and residence addresses of the 1554 participants in Nanjing, China

We obtained daily average PM air quality indices (AQIs) and concentrations published daily by the Nanjing Environmental Protection Bureau. To make our results comparable with those from other studies, we chose concentrations (μg/m^3^) in our analysis. PM_2.5_ and PM_10_ concentrations were continuously measured at nine fixed state-controlled air quality monitoring stations located in Nanjing city (Fig. 1). Data on daily ambient average temperature (in Celsius) in Nanjing over the same period were obtained from the Nanjing Regional Climate Center. Each participant was interviewed to collect information including residence address, age, height, weight, ethnicity, education, family income, cigarette smoking and alcohol consumption. Body mass index (BMI, in kg/m^2^) was calculated as weight in kilograms (kg) divided by height in meters squared (m^2^). Participants were asked about the number of days of abstinence from ejaculation. After an interview, each participant donated a semen sample for semen quality analysis.

All semen samples were collected during the second trimester of pregnancy of the participants’ spouses. Subjects were instructed to collect semen samples by masturbation into sterile plastic specimen containers in a semen collection room. Semen specimens were allowed to liquefy at 37 °C, and aliquots were analyzed at approximately 30 min after ejaculation using computer-assisted semen analysis (CASA) in accordance with guidelines of the WHO 5th Laboratory Manual for the Examination of Human Semen [32]. Semen volume was measured using a sterile serological pipette. Sperm outcomes include semen volume, sperm concentration, total sperm number, total motility, progressive motility. Additionally, motility measures are curvilinear velocity (VCL), straight-line velocity (VSL), linearity (LIN), average path velocity (VAP), wobble (WOB), straightness (STR), mean angular displacement (MAD), beat cross frequency (BCF), and amplitude of lateral head displacement (ALH). VCL indicates the average velocity of the sperm head along its curved path. VSL indicates the average straight-line velocity of the sperm head from the initial location to the last location. VAP indicates the time-averaged velocity of the sperm head moving along its average trajectory. MAD indicates the average angle of the sperm head turning along the curved path. BCF indicates the time-averaged velocity of the sperm curve trajectory across its average path. ALH indicates the swaying amplitude of the sperm head along its average trajectory (Fig. S1).

### Exposure assessment

First, we used XGeocoding software to convert the participants’ specific home address information and the locations of nine atmospheric monitoring stations in Nanjing into longitude and latitude values. Then we imported the longitude and latitude data and the average exposure values of pollutants at different stages into ArcGIS software. The location of nine atmospheric monitoring stations in Nanjing enables them to effectively monitor air pollution throughout the administrative area. On that basis, we used an inverse distance weighting (IDW) modeling method to assign PM_2.5_ and PM_10_ exposure levels for each residence address on each day using daily pollutant concentrations from air quality monitoring data between January 1st, 2014 and December 31st, 2016 [33]. IDW interpolation was used as a spatial interpolation method to model the distribution of air pollutants using data from the fixed monitoring stations. The process of spermatogenesis includes a series of complex steps including stem cell replication, meiosis, and spermiogenesis that occur over approximately 74 days in humans [34]. With several days of epididymal transit time (3-12 days) and an abstinence interval (controlled in the analysis), an exposure period of approximately 90 days (a spermatogenic cycle) is regarded as being of sufficient duration to detect effects on any stage of spermatogenesis [25]. Therefore, concentrations of PM_2.5_ and PM_10_ were calculated accordingly for the entire 90-day period and four key periods (0-9, 10-14, 15-69, 70-90 days before semen collection) preceding sampling [30].

### Statistical analysis

Basic descriptive statistics were calculated to characterize the demographic information, PM exposure, and semen quality parameters of the study population. We analyzed the associations between the air pollution variables and semen parameters using adjusted multivariable linear regression models and obtained coefficients and 95% confidence interval (CI). Given the non-normal distribution for semen volume, total sperm number, MAD, VSL, STR, ALH, and BCF, these data were converted to base-10 logarithms to meet the normality assumptions of the statistical analysis.

Selection of covariates was based on biological plausibility and their importance in the literature. We adjusted all models for age, BMI, ethnicity, education, family income, smoking status, alcohol consumption, season of sperm collection, average ambient temperature, and abstinence period. The average temperature was calculated as the mean of daily average temperature during each exposure period.

Participants were further divided into ‘normal’ and ‘abnormal’ semen quality groups based on their semen volume, sperm concentration, total sperm number and total motility. The abnormal group (*n* = 535) was defined by having at least one of the following abnormal semen parameters as defined by reference levels from WHO guidelines: semen volume < 1.5 ml, sperm concentration < 15 × 10^6^/ml, total sperm number < 39 × 10,^6^ or total motility < 40% [35]. Removing this abnormal semen parameter group left 1019 individuals as the normal group. The effect estimates were calculated for an increment of every 10 μg/m^3^ in average PM concentrations.

In addition, to better characterize exposure-response associations, we divided PM_2.5_ exposures into quintiles based on the distribution among all participants and estimated regression coefficients with the first quintile as the reference level. Effect modification by age, BMI, family income, smoking status, and drinking status was assessed by calculating product terms. A test for linear trend was conducted with the use of quintiles of the PM_2.5_ exposure variables as an ordinal variable.We also performed stratified analyses of the association between PM_2.5_ and semen quality by age (< 35 and ≥ 35 years), BMI (< 24 and ≥ 24 kg/m^2^), family income (< 100,000 yuan and ≥ 100,000 yuan), smoking status (never and former or current) and alcohol drinking status (never and former or current). R software version 4.0.2 (R Core Team R, 2020) was used to perform all statistical analyses. *P* values < 0.05 were considered significant. To address multiple testing, we also calculated the false discovery rate (FDR) using the Benjamini & Hochberg (BH) procedure and the total number of hypotheses tested was 14. All *P* values reported were two-sided.

### Role of the funding source

The funder of the study did not play any role in study design, data collection, data analysis, data interpretation, or writing of the report. The corresponding author had full access to all the data in the study and was finally responsible for the decision to submit for publication.

## Results

A flowchart of participants in the study is shown in Fig. S2. Characteristics of the entire group of 1554 fertile men and the normal (*n* = 1019) and abnormal (*n* = 535) semen quality groups are shown in Table 1. The mean age of men participating in this study was 30.9 (standard deviation [SD]: 4.2) years and two-thirds had college or higher education (66.8%). The majority of men were never smokers (62.3%) and less than half of the subjects were ever drinkers (44.5%) (Table 1).

**Table 1:** Characteristics of all fertile men participating in the study and the normal and abnormal semen quality groups

Characteristic	All participants (***n*** = 1554)	Normal semen quality group (***n*** = 1019)^**a**^	Abnormal semen quality group (***n*** = 535)^**b**^
**Age (years), n (%)**			
< 35	1279 (82.3)	867 (85.1)	412 (77.0)
≥ 35	275 (17.7)	152 (14.9)	123 (23.0)
Range	17-52	21-50	17-52
Mean (SD)	30.9 (4.2)	30.7 (4.0)	31.4 (4.4)
**BMI (kg/m**^**2**^**), n (%)**			
< 18.5	25 (1.6)	18 (1.8)	7 (1.3)
18.5 - 24.0	643 (41.4)	420 (41.2)	223 (41.7)
24.0 - 28.0	652 (42.0)	426 (41.8)	226 (42.2)
≥ 28.0	234 (15.0)	155 (15.2)	79 (14.8)
Mean (SD)	24.7 (3.3)	24.7 (3.3)	24.6 (3.2)
**Ethnicity, n (%)**			
Han	1507 (97.0)	986 (96.8)	521 (97.4)
Other	47 (3.0)	33 (3.2)	14 (2.6)
**Education, n (%)**			
Middle school and below	19 (1.2)	10 (1.0)	9 (1.7)
High school and secondary school	497 (32.0)	321 (31.5)	176 (32.9)
College degree and above	1038 (66.8)	688 (67.5)	350 (65.4)
**Family income, n (%)**			
< 100,000	558 (35.9)	359 (35.2)	199 (37.3)
100,000 - 200,000	706 (45.4)	480 (47.0)	226 (42.3)
≥ 200,000	290 (18.7)	181 (17.8)	109 (20.4)
**Smoking status, n (%)**			
Never smoker	968 (62.3)	629 (61.7)	339 (63.4)
Ever smoker	586 (37.7)	390 (38.3)	196 (36.6)
Current smoker	500 (32.2)	334 (32.8)	166 (31.0)
Former smoker	86 (5.5)	56 (5.5)	30 (5.6)
**Drinking status, n (%)**			
Never drinker	862 (55.5)	575 (56.5)	287 (53.6)
Ever drinker	692 (44.5)	444 (43.5)	248 (46.4)
Current drinker	597 (38.4)	388 (38.0)	209 (39.1)
Former drinker	95 (6.1)	56 (5.5)	39 (7.3)
**Season of sperm collection, n (%)**			
Spring	457 (29.4)	336 (33.0)	121 (22.6)
Summer	358 (23.0)	225 (22.1)	133 (24.9)
Autumn	371 (23.9)	225 (22.1)	146 (27.3)
Winter	368 (23.7)	233 (22.8)	135 (25.2)
**Days of abstinence, mean (SD)**			
< 3	375 (24.1)	176 (17.3)	199 (37.2)
3 - 5	699 (45.0)	505 (49.5)	194 (36.3)^*^
≥ 5	480 (30.9)	338 (33.2)	142 (26.5)^*^
Mean (SD)	3.9 (2.6)	4.1 (2.7)	3.6 (2.4)
**Semen volume (ml), mean (SD)**	2.7 (1.3)	2.9 (1.2)	2.3 (1.5)^*^
**Sperm concentration (10**^**6**^**/ml)**^**c**^	58.3 (37.1-84.4)	63.6 (43.0-87.7)	47.5 (25.6-76.6)^*^
**Total sperm number (10**^**6**^**)**^**c**^	142.9 (79.8-231.8)	168.0 (111.9-248.6)	81.9 (34.2-169.6)^*^
**Total motility (%)**^**c**^	56.3 (42.0-69.2)	61.8 (52.2-73.2)	36.0 (26.6-52.6)^*^

Note: *SD* standard deviation, *BMI* body mass index

^a^Group defined by semen volume ≥ 1.5 ml, sperm concentration ≥ 15 × 10^6^/ml, total sperm number ≥ 39 × 10,^6^ and total motility ≥40%

^b^Group defined by at least one abnormal semen parameter (semen volume, sperm concentration, total sperm number or total motility)

^c^Values are given as median (P25 - P75)

^*^P <  0.05 when compared with normal semen quality group

**Fig. 2:**
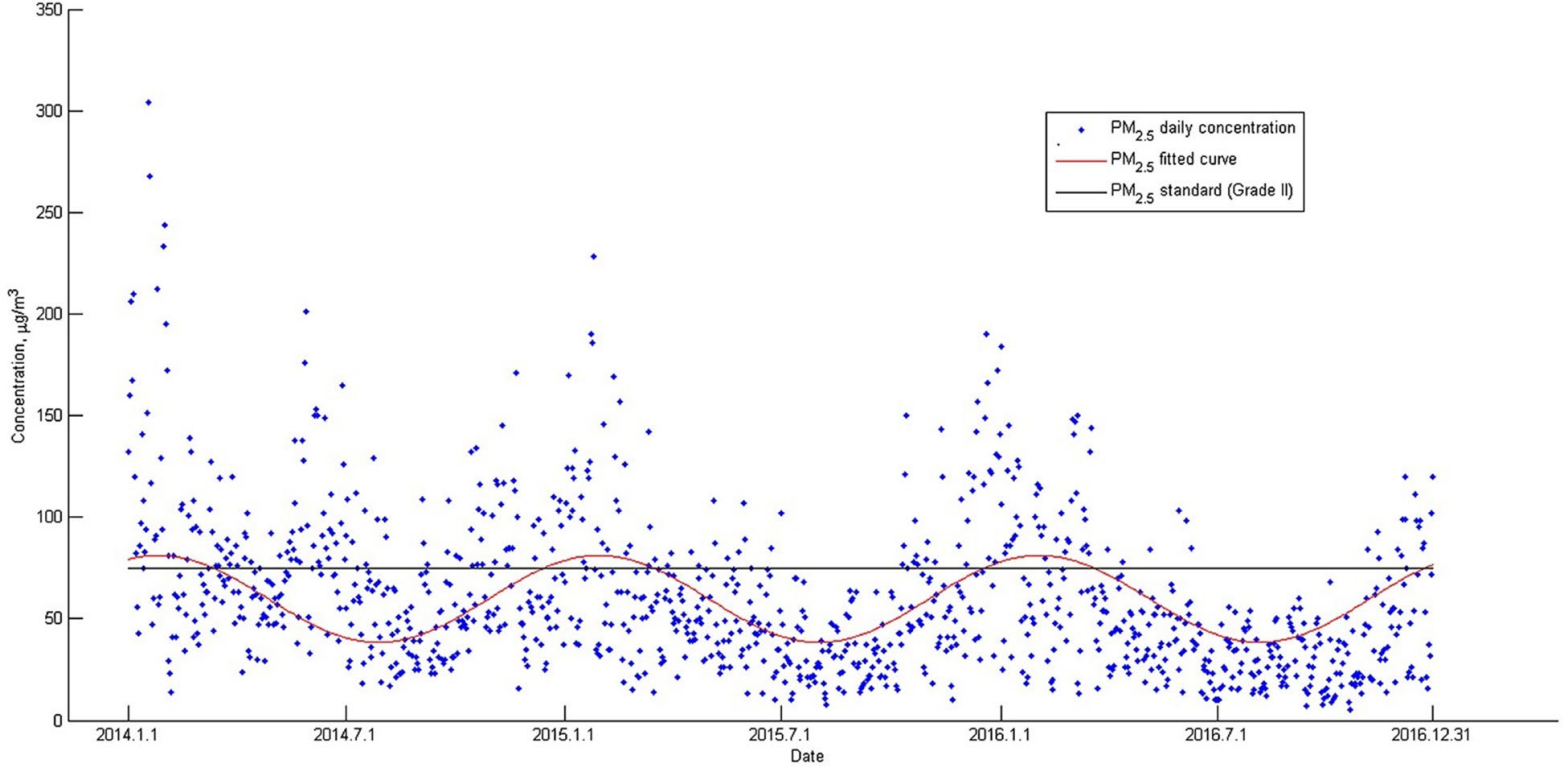
Distribution of daily PM_2.5_ in Nanjing between 2014 and 2016. The points in top and bottom graphs indicate daily PM_2.5_. The straight black line indicates Chinese 24-h standard (Grade II) for PM_2.5_ (75 μg/m^3^)

Table 2 shows the distributions of the semen parameters for all participants. The Pearson correlations (r) between the 14 semen parameters are shown in Table S1. Total motility and progressive motility were very highly correlated (r = 0.94) as were straight-line velocity (VSL) and average path velocity (VAP) (r = 0.96), linearity (LIN) and wobble (WOB) (r = 0.94) (Table S1). Fig. S3 shows average daily temperatures in Nanjing between 2014 and 2016. The daily average temperature was as high as 34.1 °C in summer and as low as − 6.6 °C in winter. Average daily concentrations of PM_2.5_ and PM_10_ are plotted in Fig. 2 and Fig. S4 respectively. The daily concentrations averaged 59.61 μg/m^3^ for PM_2.5_ and 101.77 μg/m^3^ for PM_10_ over the study period (January 1st, 2014 - December 31st, 2016, 1098 days). PM_2.5_ and PM_10_ were positively correlated (r = 0.92, *P* <  0.05). The concentrations of PM_2.5_ and PM_10_ showed clear seasonal variation with higher levels in winter (maximums of 83.44 μg/m^3^ for PM_2.5_ and 134.82 μg/m^3^ for PM_10_) than summer (minimums of 45.19 μg/m^3^ for PM_2.5_ and 72.93 μg/m^3^ for PM_10_). During the study period, PM_2.5_ was above the Chinese 24-h standard (75 μg/m^3^, Grade II) on over 26% of days; the rate of exceedance was lower for PM_10_ (16.7% of days). Table S2 gives descriptive statistics for estimated 90-days participant exposures to the two pollutants. For PM_2.5_, the average 90-days concentration was 60.80 μg/m^3^ (SD = 13.30; interquartile range, IQR = 17.9). For PM_10_, the average 90-days concentrations was 103.10 μg/m^3^ (SD = 20.50; interquartile range, IQR = 34.10).

**Table 2:** Distribution of semen parameters for the participants (*n* = 1554)

Semen parameter^**a**^	Mean (SD)	Percentile				
		10th	25th	50th	75th	90th
**Semen volume (ml)**	2.7 (1.3)	1.0	2.0	2.0	3.0	5.0
**Sperm concentration (10**^**6**^**/ml)**	62.0 (32.0)	23.3	37.1	58.3	84.4	108.0
**Total sperm number (10**^**6**^**)**	172.2 (129.9)	39.6	79.2	142.6	231.3	341.7
**Total motility (%)**	55.2 (19.3)	28.5	42.0	56.2	69.3	80.0
**Progressive motility (%)**	43.8 (16.9)	21.9	31.8	44.1	55.9	65.4
**VCL (μm/s)**	47.7 (8.9)	37.3	42.1	47.2	53.3	59.2
**VSL (μm/s)**	29.8 (6.1)	23.0	25.8	29.6	33.6	37.6
**VAP (μm/s)**	33.5 (6.2)	26.4	29.4	33.4	37.5	41.3
**BCF (Hz)**	5.1 (0.7)	4.3	4.6	5.1	5.5	6.0
**ALH (μm/s)**	3.6 (1.1)	2.4	2.9	3.6	4.3	4.9
**LIN (%)**	61.3 (7.5)	52.1	56.2	61.2	66.4	70.8
**STR (%)**	85.2 (4.1)	80.2	83.0	85.6	88.0	89.8
**WOB (%)**	70.3 (6.4)	62.6	65.7	70.2	74.6	78.3
**MAD (°)**	56.6 (7.9)	46.8	52.0	57.2	62.1	65.9

^a^*ALH* amplitude of lateral head displacement, *BCF* beat cross frequency, *LIN* linearity, *MAD* mean angular displacement, *SD* standard deviation, *STR* straightness, *VAP* average path velocity, *VCL* curvilinear velocity, *VSL* straight line velocity, *WOB* curvilinear path wobble

The results of the regression models for PM_2.5_ during 0-90, 0-9, 10-14, 15-69, 70-90 days before the date of semen examination in all participants are summarized in Table 3. For the 0-90 days exposure period, an increment of 10 μg/m^3^ in PM_2.5_ was associated with a 2.21% decrease in total motility and a 1.93% decrease in progressive motility. Inverse associations were also seen by PM_2.5_ for the sperm motion parameters VCL, VSL, VAP and ALH (all *P* <  0.05). No statistically significant association was observed for semen volume, sperm concentration, or total sperm number. For the four key periods of sperm development (0-9, 10-14, 15-69, and 70-90 days), the associations between PM_2.5_ exposure and progressive motility as well as for the motion parameters BCF were only related to the 70-90 days exposure period.

**Table 3:** Coefficients from linear regression for PM_2.5_ exposure in relation to semen parameters by exposure period (0 - 90, 0 - 9, 10 - 14, 15 - 69, 70 - 90 days) prior to semen collection in all participants (*n* = 1554) expressed as change in the parameter for a 10 μg/m^3^ increase in exposure

Semen parameter^**a**^	0-90 days		0-9 days		10-14 days		15-69 days		70-90 days	
	Coefficient (×10; 95% CI)	***P***^**b**^	Coefficient (×10; 95% CI)	***P***^**b, c**^	Coefficient (×10; 95% CI)	***P***^**b, d**^	Coefficient (× 10; 95% CI)	***P***^**b, e**^	Coefficient (×10; 95% CI)	***P***^**b, f**^
Semen volume (ml)	−0.003 (−0.025, 0.018)	0.965	−0.004 (−0.018, 0.010)	0.980	0.002 (−0.013, 0.010)	0.975	−0.002 (−0.023, 0.019)	0.851	0.009 (−0.007, 0.025)	0.324
Concentration (10^6^/ml)	0.178 (−1.342, 1.698)	0.965	−0.127 (−1.105, 0.802)	0.980	−0.333 (−1.136, 0.471)	0.975	1.363 (−0.142, 2.868)	0.152	−0.496 (−1.625, 0.633)	0.419
Total sperm number (10^6^)	0.001 (−0.039, 0.041)	0.965	−0.004 (−0.030, 0.022)	0.980	−0.011 (−0.032, 0.011)	0.975	0.025 (−0.015, 0.065)	0.372	0.003 (−0.033, 0.027)	0.834
Total motility (%)	**−2.211 (−3.121, −1.301)**	**< 0.001**	−0.194 (−0.782, 0.394)	0.980	−0.216 (−0.698, 0.267)	0.975	−0.981 (−1.886, −0.077)	0.094	−0.770 (−1.451, −0.090)	0.075
Progressive motility (%)	**−1.925 (−2.720, −1.131)**	**< 0.001**	−0.135 (−0.650, 0.380)	0.980	−0.090 (−0.512, 0.332)	0.975	−0.893 (−1.685, −0.102)	0.094	**−0.828 (−1.421, −0.234)**	**0.029**
VCL (μm/s)	**−1.054 (−1.467, −0.640)**	**< 0.001**	−0.202 (−0.470, 0.066)	0.841	−0.011 (−0.208, 0.231)	0.989	**−0.714 (−1.125, −0.302)**	**0.009**	−0.306 (−0.613, −0.001)	0.089
VSL (μm/s)	**−0.022 (−0.032, −0.011)**	**< 0.001**	−0.005 (−0.011, −0.002)	0.841	−0.001 (−0.006, 0.005)	0.975	**−0.014 (−0.025, −0.004)**	**0.038**	**−0.013 (−0.021, −0.006)**	**0.004**
VAP (μm/s)	**−0.714 (−1.000, −0.428)**	**< 0.001**	−0.165 (−0.350, 0.020)	0.841	−0.016 (−0.135, 0.168)	0.975	**−0.469 (−0.752, −0.185)**	**0.009**	**−0.389 (−0.598, −0.371)**	**0.004**
BCF (Hz)	0.0004 (−0.003, 0.010)	0.510	−0.0004 (−0.004, 0.004)	0.980	−0.001 (−0.004, 0.002)	0.975	0.003 (−0.004, 0.009)	0.620	**0.006 (0.002, 0.011)**	**0.031**
ALH (μm/s)	**−0.023 (−0.037, −0.008)**	**0.005**	0.004 (−0.005, 0.014)	0.980	0.0001 (−0.008, 0.008)	0.989	−0.014 (−0.029, 0.0001)	0.112	−0.006 (−0.017, 0.004)	0.312
LIN (%)	−0.018 (−0.377, 0.340)	0.965	−0.003 (−0.235, 0.229)	0.980	0.071 (−0.119, 0.262)	0.975	−0.071 (−0.428, 0.285)	0.851	−0.264 (−0.528, −0.0002)	0.089
STR (%)	−0.001 (−0.003, 0.002)	0.965	0.00002 (−0.002, 0.002)	0.980	0.001 (−0.0004, 0.002)	0.975	0.0003 (−0.003, 0.002)	0.851	−0.002 (−0.004, −0.0001)	0.086
WOB (%)	−0.005 (−0.301, 0.310)	0.965	−0.009 (−0.207, 0.189)	0.980	0.019 (−0.143, 0.181)	0.975	−0.035 (−0.338, 0.268)	0.851	−0.217 (−0.443, −0.008)	0.091
MAD (°)	0.004 (−0.009, 0.010)	0.965	−0.002 (−0.008, 0.005)	0.980	−0.002 (−0.007, 0.003)	0.975	0.001 (−0.008, 0.011)	0.851	0.006 (−0.002, 0.013)	0.174

^a^*ALH* amplitude of lateral head displacement, *BCF* beat cross frequency, *LIN* linearity, *MAD* mean angular displacement, *PM*_*2.5*_ particulate matter with aerodynamic less than 2.5 μm, *CI* confidence interval, *STR* straightness, *VAP* average path velocity, *VCL* curvilinear velocity, *VSL* straight line velocity, *WOB* curvilinear path wobble

^b^Results were adjusted for age, BMI, ethnicity, education, smoking status, drinking status, family income, abstinence period, season, and temperature. *P* value for adjusting FDR using the Benjamini & Hochberg procedure

^c^Ambient particulate matter exposure of 10-14 days, 15-69 days, 70-90 days were also adjusted

^d^Ambient particulate matter exposure of 0-9 days, 15-69 days, 79-90 days were also adjusted

^e^Ambient particulate matter exposure of 0-9 days, 10-14 days, 70-90 days was also adjusted

^f^Ambient particulate matter exposure of 0-9 days, 10-14 days, 15-69 days was also adjusted

Table S3 presents the results of regression models of PM_10_ exposure and semen quality in all subjects. For the entire 0-90 days exposure period, PM_10_ exposure were significantly associated with the velocity parameters VCL and VAP, both inverse. Divided into four key periods of sperm development (0-9, 10-14, 15-69, and 70-90 days), these statistically significant inverse associations were limited to 70-90 days prior to semen collection (Table S3). The analyses of PM_2.5_ and PM_10_ in normal semen quality males during the entire 0-90 days exposure period generally yielded similar findings to the entire dataset (Tables S4 and S5). As for PM_2.5_, the coefficients of total motility and progressive motility were − 1.444 and − 1.370 respectively in the normal group. But the associations became no statistically significant after the FDR adjustment in the abnormal group.

Table 4 shows the results of exposure-response analyses using quintiles of PM_2.5_ in the 0-90 days before semen collection and semen quality in all participants. For all participants, PM_2.5_ exposure was inversely associated with total motility and progressive motility. The coefficient of total motility of the highest quintile of PM_2.5_ concentration compared with the lowest was − 8.42 (95%CI: − 11.98, − 4.86; *P*-trend < 0.001) for PM_2.5_ exposure (Table 4). In normal semen quality group, the coefficient of total motility of the highest quintile of PM_2.5_ exposure compared with the lowest was − 5.56 (95%CI: − 8.79, − 2.33; *P*-trend = 0.006) (Table S6). It seemed that the associations were linear as suggested by monotonic trends across quintiles of PM_2.5_ exposure *(P*-trend < 0.05). In abnormal semen quality group, the coefficient of total motility of the highest quintile of PM_2.5_ exposure compared with the lowest was of − 7.35 (95%CI: − 13.16, − 1.55; *P*-trend = 0.546) (Table S7). It indicated that the association between total motility and PM_2.5_ exposure was nonlinear for abnormal semen quality group.

**Table 4:** Coefficients (95% CIs) from linear regression of PM_2.5_ exposure during 0 - 90 days before semen collection in relation to semen parameters in all participants (*n* = 1554)

Semen parameter^**a**^	Quintile of PM_**2.5**_ exposure (range)					***P*** trend^**b**^
	Q1 (28.7-49.6)	Q2 (49.7-57.0)	Q3 (57.1-64.8)	Q4 (64.9-74.0)	Q5 (74.1-92.1)	
**Semen volume (ml)**	0 (reference)	0.005 (−0.07, 0.08)	−0.04 (−0.13, 0.04)	−0.07 (−0.17, 0.01)	−0.03 (−0.11, 0.06)	0.583
**Sperm concentration (10**^**6**^**/ml)**	0 (reference)	0.12 (−5.11, 5.36))	2.53 (−3.33, 8.39)	−2.18 (−8.18, 3.82)	−0.96 (−6.92, 5.00)	0.862
**Total sperm number (10**^**6**^**)**	0 (reference)	0.03 (−0.11, 0.17)	0.06 (−0.10, 0.22)	−0.07 (−0.24, 0.09)	−0.04 (−0.21, 0.12)	0.583
**Total motility (%)**	0 (reference)	**−6.21 (−9.33, −3.08)**	**−4.98 (−8.48, −1.48)**	**−7.00 (−10.58, −3.41)**	**−8.42 (−11.98, −4.86)**	**< 0.001**
**Progressive motility (%)**	0 (reference)	**−5.04 (−7.76, −2.31)**	**−3.74 (−6.79, −0.69)**	**−4.69 (−7.82, −1.57)**	**−7.41 (−10.52, −4.30)**	**< 0.001**
**VCL (**μm**/s)**	0 (reference)	−0.03 (−1.46, 1.40)	−1.38 (−2.98, 0.22)	−1.18 (−2.82, 0.46)	**−3.24 (−4.82, −1.61)**	**< 0.001**
**VSL (μm/s)**	0 (reference)	−0.005 (−0.04, 0.03)	−0.01 (−0.05, 0.03)	−0.01 (−0.05, 0.03)	**−0.07 (−0.11, −0.03)**	**< 0.001**
**VAP (μm/s)**	0 (reference)	−0.21 (−1.20, 0.77)	−0.37 (−1.47, 0.73)	−0.47 (−1.60, 0.66)	**−2.27 (−3.39, −1.15)**	**< 0.001**
**BCF (Hz)**	0 (reference)	−0.002 (−0.02, 0.02)	−0.02 (−0.04, 0.003)	**−0.03 (−0.06, −0.01)**	0.01 (−0.01, 0.04)	0.611
**ALH (μm/s)**	0 (reference)	0.01 (−0.04, 0.06)	−0.02 (−0.07, 0.03)	−0.03 (−0.09, 0.02)	**−0.06 (−0.11, −0.003)**	**0.028**
**LIN (%)**	0 (reference)	−0.46 (−1.69, 0.76)	0.78 (−0.59, 2.16)	0.83 (−0.58, 2.24)	−0.37 (−1.77, 1.03)	0.971
**STR (%)**	0 (reference)	0.001 (−0.01, 0.01)	0.005 (−0.004, 0.01)	0.01 (−0.01, 0.02)	−0.003 (−0.01, 0.01)	0.862
**WOB (%)**	0 (reference)	−0.42 (−1.46, 0.63)	0.56 (−0.61, 1.73)	0.61 (−0.59, 1.81)	−0.22 (−1.42, 0.97)	0.986
**MAD (°)**	0 (reference)	−0.0005 (−0.03, 0.03)	−0.01 (−0.05, 0.02)	−0.01 (−0.03, 0.04)	0.002 (−0.03, 0.04)	0.971

The coefficients and 95% CIs were estimated using a linear model, adjusting for age, BMI, ethnicity, education, family income, smoking status, drinking status, abstinence period, season, and temperature. Natural log transformation was applied for some sperm parameters

^a^*ALH* amplitude of lateral head displacement, *BCF* beat cross frequency, *CI* confidence interval, *LIN* linearity, *MAD* mean angular displacement, *PM*_*2.5*_ particulate matter with aerodynamic less than 2.5 μm, *STR* straightness, *VAP* average path velocity, *VCL* curvilinear velocity, *VSL* straight line velocity, *WOB* curvilinear path wobble

^b^*P* trend value for adjusting FDR using the Benjamini & Hochberg procedure

The results of the analyses of PM_2.5_ in relation to total sperm motility stratified by age, BMI, family income, smoking, and alcohol drinking are summarized in Table S8. The effect estimates were calculated for an increment of every 10 μg/m^3^ in average PM_2.5_ concentrations. Inverse associations were found in all subgroups of BMI, family income (*P* <  0.05). For participants whose age were over 35 years, who were former or current smokers and who were former or current drinkers, there were no obvious correlation between PM_2.5_ exposure and total motility (*P* = 0.180, *P* = 0.053 and *P* = 0.195).

## Discussion

As far as we know, there are few studies investigating the effects of PM exposure on semen quality in a such large population of men known to be fertile (Table S9). Taking into account potential confounding factors including season and temperature, we found that PM_2.5_ exposure was negatively associated with sperm motility, both total and progressive. The inverse associations with PM_2.5_ exposure were generally stronger during 70-90 lag days than those associations in the other three periods examined, which indicated that PM_2.5_ exposure might reduce human semen quality by generally influencing the early stage of sperm development and sperm motility. PM_2.5_ carries various environmental pollutants such as heavy metals which interfere with germ cell function and affect gene expression [36]. Spermatogenesis goes through three stages: proliferative phase, meiotic phase, and spermatogenic phase. During the proliferative phase, the gene expression caused by the contaminant is transmitted. We identified statistically significant findings but we do not know if these have clinical significance. We note that although there are men in this population with sperm parameters that meet a WHO definition of “abnormal”, all of these men are fertile. Although we do not have data to address this question, it is possible that these differences in sperm parameters could have subtle effects on fertility, such as time to pregnancy. The associations between PM_10_ and sperm motility were weaker compared with those for PM_2.5_, which further implicates PM_2.5_ specifically as a reproductive toxicant. No significant association was found between PM and sperm concentration.

Motility parameters have been reported to be sensitive biomarkers of human reproductive toxicity [37]. Fertility assessment of men is generally dependent on the quality assessment of semen by conventional parameters such as motility, concentration, and morphology of spermatozoa. Sperm motility is considered one of the most important sperm functions that affect natural conception. Reduced sperm motility causes about 18% of male infertility and infertility cases [38]. In our study, we found that PM_2.5_ was consistently associated with decreased sperm total motility and progressive motility, but not sperm concentration. And the coefficients in total population were higher than in the normal group. It indicated that the general population was much more sensitive to PM_2.5_ exposure to some extent. The inverse associations prompted reduced motility caused by PM_2.5_ exposure may result in infertility but not absolutely. Reduced motility would also affect the time to pregnancy [39], which needs to be collected and analyzed in further study. Similar results, a negative correlation between PM_2.5_ and sperm motility, were reported by Hammoud et al. [40]. Lao et al. [28] did not find significant associations between PM_2.5_ exposure and sperm motility in reproductive-age men. Selevan et al. [25] examined the relationship between air pollution levels and VSL, VCL, and LIN, and found that medium levels of air pollution were negatively associated with VCL, but positively associated with LIN. Wu et al. [31], in a study of 1759 infertile men, reported that decreased sperm concentration and count, but not sperm motility, were associated with PM_2.5_. A recent study explored the association between PM exposure and semen quality in a cohort of undergraduate students and suggested that PM_10_ but not PM_2.5_ is associated with semen quality [41]. These conflicting research results may be due to geographic or racial differences and the differences in study designs and methods. In particular, exposure concentrations of pollutants vary from region to region (Table S8). Thus, although they are the same contaminant, their effect on semen quality varies depending on the amount of individual exposure. The participants of most previous studies were infertile men [31, 42, 43], which may cause selection bias. While in our study, participants were fertile men which could better represent the general population. Besides, different measures of individual exposure also contribute to the discrepancy. Lao et al. [28] used a spatiotemporal model with high resolution (1 × 1 km) to estimate individual exposure of PM_2.5_. Some other studies estimated air pollution exposure at the community level [19, 25]. It may mask exposure variation and cause misclassification.

Smoking has been reported to be detrimental to semen quality. Sokol et al. found that an increase in O_3_ levels was associated with a decline in sperm quality, but they did not look at the effect modifications of cigarette smoking on pollution and semen quality [29]. Considering that the smoking effect may operate concurrently in the similar pathways [39], we further analyzed the associations between PM_2.5_ exposure and semen quality by smoking status. We found significant effect modifications by smoking in the association between PM_2.5_ and semen quality. Furthermore, we noted that our findings were seen across age subgroups and drinking status subgroups.

As one of the most significant characteristics related to the fertilizing ability of spermatozoa, motility reflects their viability and structural integrity [44]. However, the biological mechanisms behind the association between PM exposure and decreased sperm motility have yet to be determined. Previous investigations have suggested that PM exposure is probably associated with increased oxidative stress due to decreased antioxidant defenses or excess reactive oxygen species (ROS) production. Oxidative stress performs an essential role to trigger cellular pathological process including proliferation, inflammation, and apoptosis [45]. PM_2.5_ component species include elemental carbon, organic compounds like polycyclic aromatic hydrocarbons (PAHs), and heavy metals [46]. PM_2.5_ is mainly deposited within the distal alveoli after inhalation [42]. Such PM_2.5_ exposure will result in ROS generation which then leads to systematical oxidative stress and cell impairment. An animal study has reported that Sertoli cells (SCs) would produce a large amount of ROS after exposure to PM_2.5_. The oxidative stress damage in cells resulted in activation of the mitogen-activated protein kinases (MAPK) pathway, increasing SCs apoptosis, then destroying the integrity of the blood-testis barrier, finally causing the quality of semen [47]. Moreover, PAHs and multiple trace elements in PM_2.5_ might contribute to poor sperm quality. Human and animal studies have suggested possible associations between PAH exposure and male reproductive function [48, 49]. Izawa et al. [50] demonstrated inverse associations between PAH exposures and sperm production, sperm abnormalities and sperm motility in an animal study. Our previous study suggested that PAH exposure contributed to decreased semen quality in a Chinese population [51]. Adverse influences of metals such as cadmium and lead on spermatogenesis have been demonstrated [52]. Further studies are warranted to elucidate the underlying mechanism as well as the specific components of PM_2.5_ that may be driving associations.

Some limitations need to be addressed. As in most studies of health effects of air pollution, we did not measure exposure directly at the individual level. It is not accurate to substitute the average exposure of a region for that of an individual. And we didn’t rule out the influence of individual time-activities. However, sampling air pollution at the individual level is not realistic. Moreover, there is no clear exposure marker to characterize individual exposure levels. Secondly, we estimated ambient PM_2.5_ and PM_10_ using outdoor air monitors, but indoor environments and time-activity patterns can also influence individual and population level exposures. Thus, individual exposure is underestimated. In part, it may weaken the link between ambient particulate matter exposure and semen quality. Thirdly, the fertile men delivered only one semen sample each. However, a previous study has shown that while a single semen sample may not be adequate for clinical diagnosis of infertility, that it should suffice for studies aimed at identifying average differences in semen quality between individuals [53]. The associations between PM_2.5_ and sperm motility both in men with all normal sperm parameters as well as in men with at least one abnormal sperm parameter provide additional evidence that PM_2.5_ influences sperm motility across the population as a whole. Besides, we did not have the urine or blood sample to detect other exposure metabolities like endocrine disruptors.

This study has many strengths. One unique feature is that we examined representative samples from a population known to be fertile rather than the infertile populations from infertility clinics or men of unknown fertility as in most previous studies [31, 54]. When evaluating the effects of air pollution on semen quality properly, it is important to control for confounding factors [28]. We had information on various potential confounders in our study. In addition, we used the IDW interpolation to estimate individual air pollution exposures. Some previous studies estimated air pollution exposure at the community level, which could cause misclassification and mask exposure variation [55]. Further, we assessed a wide range of 14 semen parameters to investigate the associations between PM exposure and semen quality in a more comprehensive manner than in most studies.

In conclusion, a robust association was found between exposure to PM_2.5_ in specific window (70-90 days prior to ejaculation) and reduced sperm motility measures in fertile men. We did not see significant associations between PM_2.5_ and sperm concentration or count. These results indicate that PM_2.5_ exposure does not reduce the number of sperm produced but does impact its functionality which could reduce the ability to fertilize the ovum. Meanwhile, PM_10_ plays a less important role than PM_2.5_ in the relationship between PM exposure and sperm motility. This suggests that PM_2.5_ is a more meaningful reproductive toxicant.

## Supplementary Information

**Additional file 1: Figure S1.** Sperm kinematic parameters measured by computer assisted semen analysis (CASA). ALH, amplitude of lateral head displacement; BCF, beat cross frequency; LIN, linearity; MAD, mean angular displacement; STR, straightness; VAP, average path velocity; VCL, curvilinear velocity; VSL, straight line velocity; WOB, curvilinear path wobble. **Figure S2.** Flowchart of participants in the study. Normal semen quality group defined by semen volume ≥ 1.5 ml, sperm concentration ≥ 15 × 10^6^/ml, total sperm number ≥ 39 × 10,^6^ and total motility ≥40%. Abnormal semen quality group defined by at least one abnormal semen parameters (semen volume, sperm concentration, total sperm number or sperm motility). **Figure S3.** Distribution of daily temperatures in Nanjing between 2014 and 2016. The points in top and bottom graphs indicate daily temperatures. **Figure S4.** Distribution of daily PM_10_ in Nanjing between 2014 and 2016. The points in top and bottom graphs indicate daily PM_10_. The straight black line indicates Chinese 24-h standard (Grade II) for PM_10_ (150 μg/m^3^). **Table S1.** Coefficient of correlation between the semen parameters. **Table S2.** Distribution of air pollutant exposure for study subjects. **Table S3.** Coefficients from linear regression for PM_10_ exposure in relation to semen parameters by exposure period (0-90, 0-9, 10-14, 15-69, 70-90 days) prior to semen collection in all participants (*n* = 1554) expressed as change in the parameter for a 10 μg/m^3^ increase in exposure. **Table S4.** Coefficients from linear regression for PM_2.5_ exposure in relation to semen parameters by exposure period (0-90, 0-9, 10-14, 15-69, 70-90 days) prior to semen collection in normal and abnormal semen parameters groups expressed as change in the parameter for a 10 μg/m^3^ increase in exposure. **Table S5**. Coefficients from linear regression for PM_10_ exposure in relation to semen parameters by exposure period (0-90, 0-9, 10-14, 15-69, 70-90 days) prior to semen collection in normal and abnormal semen parameters groups expressed as change in the parameter for a 10 μg/m^3^ increase in exposure. **Table S6.** Coefficients (95% CIs) from linear regression of PM_2.5_ exposure during 0-90 days before semen collection in relation to sperm parameters in normal semen parameters group expressed as change in the parameter for a 10 μg/m^3^ increase in exposure. **Table S7.** Coefficients (95% CIs) from linear regression of PM_2.5_ exposure during 0-90 days before semen collection in relation to sperm parameters in abnormal semen parameters group expressed as change in the parameter for a 10 μg/m^3^ increase in exposure. **Table S8.** Coefficients from linear regression for PM_2.5_ exposure in relation to total sperm motility by categories of age, BMI, income, cigarette smoking and alcohol drinking expressed as change in the parameter for a 10 μg/m^3^ increase in exposure. **Table S9.** Characteristics and main results of previous studies examined the association between PM and semen quality.

## Data Availability

The datasets used and/or analysed during the current study are available from the corresponding author on reasonable request.

## References

[1] Mascarenhas MN, Flaxman SR, Boerma T, Vanderpoel S, Stevens GA (2012). National, regional, and global trends in infertility prevalence since 1990: a systematic analysis of 277 health surveys. PLoS Med.

[2] Wang X, Tian X, Ye B, Zhang Y, Zhang X, Huang S, Li C, Wu S, Li R, Zou Y (2020). The association between ambient temperature and sperm quality in Wuhan, China. Environ Health.

[3] Mao H, Feng L, Yang WX (2017). Environmental factors contributed to circannual rhythm of semen quality. Chronobiol Int.

[4] Mendiola J, Jorgensen N, Andersson AM, Stahlhut RW, Liu F, Swan SH (2014). Reproductive parameters in young men living in Rochester, New York. Fertil Steril.

[5] Jurewicz J, Hanke W, Radwan M, Bonde JP (2009). Environmental factors and semen quality. Int J Occup Med Environ Health.

[6] Li CJ, Yeh CY, Chen RY, Tzeng CR, Han BC, Chien LC (2015). Biomonitoring of blood heavy metals and reproductive hormone level related to low semen quality. J Hazard Mater.

[7] Gulland A (2002). Air pollution responsible for 600 000 premature deaths worldwide. BMJ.

[8] Cheng Z, Wang S, Jiang J, Fu Q, Chen C, Xu B, Yu J, Fu X, Hao J (2013). Long-term trend of haze pollution and impact of particulate matter in the Yangtze River Delta, China. Environ Pollut.

[9] Brook RD, Rajagopalan S, Pope CA, Brook JR, Bhatnagar A, Diez-Roux AV, Holguin F, Hong Y, Luepker RV, Mittleman MA (2010). Particulate matter air pollution and cardiovascular disease: An update to the scientific statement from the American Heart Association. Circulation.

[10] Chen J, Hoek G (2020). Long-term exposure to PM and all-cause and cause-specific mortality: a systematic review and meta-analysis. Environ Int.

[11] Landrigan PJ, Fuller R, Acosta NJR, Adeyi O, Arnold R, Basu NN, Baldé AB, Bertollini R, Bose-O'Reilly S, Boufford JI (2018). The lancet commission on pollution and health. Lancet.

[12] Turner MC, Andersen ZJ, Baccarelli A, Diver WR, Gapstur SM, Pope CA 3rd, et al. Outdoor air pollution and cancer: an overview of the current evidence and public health recommendations. CA Cancer J Clin. 2020;70:460–47. 10.3322/caac.21632 PMC7904962 32964460

[13] Yang D, Ma M, Zhou W, Yang B, Xiao C (2017). Inhibition of miR-32 activity promoted EMT induced by PM2.5 exposure through the modulation of the Smad1-mediated signaling pathways in lung cancer cells. Chemosphere.

[14] Pedersen M, Giorgis-Allemand L, Bernard C, Aguilera I, Andersen AM, Ballester F, Beelen RM, Chatzi L, Cirach M, Danileviciute A (2013). Ambient air pollution and low birthweight: a European cohort study (ESCAPE). Lancet Respir Med.

[15] Grigg J (2013). Effects of air pollution on fetal development-more than low birthweight?. Lancet Respir Med.

[16] Somers CM (2011). Ambient air pollution exposure and damage to male gametes: human studies and in situ 'sentinel' animal experiments. Syst Biol Reprod Med.

[17] Kim KH, Kabir E, Kabir S (2015). A review on the human health impact of airborne particulate matter. Environ Int.

[18] Pires A, de Melo EN, Mauad T, Nascimento Saldiva PH, de Siqueira Bueno HM (2011). Pre- and postnatal exposure to ambient levels of urban particulate matter (PM(2.5)) affects mice spermatogenesis. Inhal Toxicol.

[19] Zhou N, Cui Z, Yang S, Han X, Chen G, Zhou Z, Zhai C, Ma M, Li L, Cai M (2014). Air pollution and decreased semen quality: a comparative study of Chongqing urban and rural areas. Environ Pollut.

[20] Carré J, Gatimel N, Moreau J, Parinaud J, Léandri R (2017). Does air pollution play a role in infertility?: a systematic review. Environ Health.

[21] De Rosa M, Zarrilli S, Paesano L, Carbone U, Boggia B, Petretta M, Maisto A, Cimmino F, Puca G, Colao A (2003). Traffic pollutants affect fertility in men. Hum Reprod.

[22] Deng Z, Chen F, Zhang M, Lan L, Qiao Z, Cui Y, An J, Wang N, Fan Z, Zhao X (2016). Association between air pollution and sperm quality: a systematic review and meta-analysis. Environ Pollut.

[23] Guven A, Kayikci A, Cam K, Arbak P, Balbay O, Cam M (2008). Alterations in semen parameters of toll collectors working at motorways: does diesel exposure induce detrimental effects on semen?. Andrologia.

[24] Rubes J, Selevan SG, Evenson DP, Zudova D, Vozdova M, Zudova Z, Robbins WA, Perreault SD (2005). Episodic air pollution is associated with increased DNA fragmentation in human sperm without other changes in semen quality. Hum Reprod.

[25] Selevan SG, Borkovec L, Slott VL, Zudova Z, Rubes J, Evenson DP, Perreault SD (2000). Semen quality and reproductive health of young Czech men exposed to seasonal air pollution. Environ Health Perspect.

[26] Johnson L, Falk GU, Suggs LC, Henderson DJ, Spoede GE, Brown SW, McGowen TA, Meguerditchian H, Barnard JJ (1997). Heterotopic transplantation as a model to study the regulation of spermatogenesis; some histomorphological considerations about sperm decline in man. Contracept Fertil Sex.

[27] Radwan M, Jurewicz J, Polanska K, Sobala W, Radwan P, Bochenek M, Hanke W (2016). Exposure to ambient air pollution--does it affect semen quality and the level of reproductive hormones?. Ann Hum Biol.

[28] Lao XQ, Zhang Z, Lau AKH, Chan TC, Chuang YC, Chan J, Lin C, Guo C, Jiang WK, Tam T (2018). Exposure to ambient fine particulate matter and semen quality in Taiwan. Occup Environ Med.

[29] Sokol RZ, Kraft P, Fowler IM, Mamet R, Kim E, Berhane KT (2006). Exposure to environmental ozone alters semen quality. Environ Health Perspect.

[30] Hansen C, Luben TJ, Sacks JD, Olshan A, Jeffay S, Strader L, Perreault SD (2010). The effect of ambient air pollution on sperm quality. Environ Health Perspect.

[31] Wu L, Jin L, Shi T, Zhang B, Zhou Y, Zhou T, Bao W, Xiang H, Zuo Y, Li G (2017). Association between ambient particulate matter exposure and semen quality in Wuhan, China. Environ Int.

[32] Sharma R, Harlev A, Agarwal A, Esteves SC (2016). Cigarette smoking and semen quality: a new Meta-analysis examining the effect of the 2010 World Health Organization Laboratory methods for the examination of human semen. Eur Urol.

[33] Brauer M, Lencar C, Tamburic L, Koehoorn M, Demers P, Karr C (2008). A cohort study of traffic-related air pollution impacts on birth outcomes. Environ Health Perspect.

[34] Clermont Y (1963). The cycle of the seminiferous epithelium in man. Am J Anat.

[35] Cooper TG, Noonan E, von Eckardstein S, Auger J, Baker HW, Behre HM, Haugen TB, Kruger T, Wang C, Mbizvo MT (2010). World Health Organization reference values for human semen characteristics. Hum Reprod Update.

[36] Jenardhanan P, Panneerselvam M, Mathur PP (2016). Effect of environmental contaminants on spermatogenesis. Semin Cell Dev Biol.

[37] Kwack SJ, Lee BM (2015). Comparative cytotoxicity and sperm motility using a computer-aided sperm analysis system (CASA) for isomers of Phthalic acid, a common final metabolite of phthalates. J Toxicol Environ Health A.

[38] Khosronezhad N, Hosseinzadeh Colagar A, Mortazavi SM (2015). The Nsun7 (A11337)-deletion mutation, causes reduction of its protein rate and associated with sperm motility defect in infertile men. J Assist Reprod Genet.

[39] Tang Q, Pan F, Wu X, Nichols CE, Wang X, Xia Y, London SJ, Wu W (2019). Semen quality and cigarette smoking in a cohort of healthy fertile men. Environ Epidemiol.

[40] Hammoud A, Carrell DT, Gibson M, Sanderson M, Parker-Jones K, Peterson CM (2010). Decreased sperm motility is associated with air pollution in Salt Lake City. Fertil Steril.

[41] Zhou N, Jiang C, Chen Q, Yang H, Wang X, Zou P, Sun L, Liu J, Li L, Li L (2018). Exposures to Atmospheric PM10 and PM10-2.5 Affect Male Semen Quality: Results of MARHCS Study. Environ Sci Technol.

[42] Guan Q, Chen S, Wang B, Dou X, Lu Y, Liang J, Ni R, Yang C, Wang H, Baktash MB (2020). Effects of particulate matter exposure on semen quality: a retrospective cohort study. Ecotoxicol Environ Saf.

[43] Tian XJ, Wang XC, Ye B, Li CL, Zhang Y, Ma L (2017). The effects of exposure to ozone on sperm quality in Wuhan. Zhonghua Yu Fang Yi Xue Za Zhi.

[44] Nagy A, Polichronopoulos T, Gaspardy A, Solti L, Cseh S (2015). Correlation between bull fertility and sperm cell velocity parameters generated by computer-assisted semen analysis. Acta Vet Hung.

[45] Saffari M, Koenig HG, Pakpour AH, Sanaeinasab H, Jahan HR, Sehlo MG (2014). Personal hygiene among military personnel: developing and testing a self-administered scale. Environ Health Prev Med.

[46] White AJ, Kresovich JK, Keller JP, Xu Z, Kaufman JD, Weinberg CR, Taylor JA, Sandler DP (2019). Air pollution, particulate matter composition and methylation-based biologic age. Environ Int.

[47] Liu B, Shen LJ, Zhao TX, Sun M, Wang JK, Long CL, He DW, Lin T, Wu SD, Wei GH (2020). Automobile exhaust-derived PM(2.5) induces blood-testis barrier damage through ROS-MAPK-Nrf2 pathway in sertoli cells of rats. Ecotoxicol Environ Saf.

[48] Yang P, Chen D, Wang YX, Zhang L, Huang LL, Lu WQ, Zeng Q (2020). Mediation of association between polycyclic aromatic hydrocarbon exposure and semen quality by spermatogenesis-related microRNAs: a pilot study in an infertility clinic. J Hazard Mater.

[49] Jeng HA, Yu L (2008). Alteration of sperm quality and hormone levels by polycyclic aromatic hydrocarbons on airborne particulate particles. J Environ Sci Health A Tox Hazard Subst Environ Eng.

[50] Izawa H, Kohara M, Watanabe G, Taya K, Sagai M (2007). Effects of diesel exhaust particles on the male reproductive system in strains of mice with different aryl hydrocarbon receptor responsiveness. J Reprod Dev.

[51] Xia Y, Zhu P, Han Y, Lu C, Wang S, Gu A, Fu G, Zhao R, Song L, Wang X (2009). Urinary metabolites of polycyclic aromatic hydrocarbons in relation to idiopathic male infertility. Hum Reprod.

[52] Pant N, Kumar G, Upadhyay AD, Patel DK, Gupta YK, Chaturvedi PK (2014). Reproductive toxicity of lead, cadmium, and phthalate exposure in men. Environ Sci Pollut Res Int.

[53] Chiu YH, Edifor R, Rosner BA, Nassan FL, Gaskins AJ, Minguez-Alarcon L, Williams PL, Tanrikut C, Hauser R, Chavarro JE (2017). What does a single semen sample tell you? Implications for male factor infertility research. Am J Epidemiol.

[54] Yang P, Wang YX, Chen YJ, Sun L, Li J, Liu C, Huang Z, Lu WQ, Zeng Q (2017). Urinary polycyclic aromatic hydrocarbon metabolites and human semen quality in China. Environ Sci Technol.

[55] Jurewicz J, Radwan M, Sobala W, Polanska K, Radwan P, Jakubowski L, Ulanska A, Hanke W (2015). The relationship between exposure to air pollution and sperm disomy. Environ Mol Mutagen.

