# Supplementary material for: Association between ambient particulate matter exposure and semen quality in fertile men
Source: Environ Health. 2022 Jan 16;21:16. doi: 10.1186/s12940-022-00831-5 (PMC8762955; doi:10.1186/s12940-022-00831-5)
Supplement: Supplementary file 1 — Additional file 1: Figure S1. Sperm kinematic parameters measured by computer assisted semen analysis (CASA). ALH, amplitude of lateral head displacement; BCF, beat cross frequency; LIN, linearity; MAD, mean angular displacement; STR, straightness; VAP, average path velocity; VCL, curvilinear velocity; VSL, straight line velocity; WOB, curvilinear path wobble. Figure S2. Flowchart of participants in the study. Normal semen quality group defined by semen volume ≥ 1.5 ml, sperm concentration ≥ 15 × 106/ml, total sperm number ≥ 39 × 10,6 and total motility ≥40%. Abnormal semen quality group defined by at least one abnormal semen parameters (semen volume, sperm concentration, total sperm number or sperm motility). Figure S3. Distribution of daily temperatures in Nanjing between 2014 and 2016. The points in top and bottom graphs indicate daily temperatures. Figure S4. Distribution of daily PM10 in Nanjing between 2014 and 2016. The points in top and bottom graphs indicate daily PM10. The straight black line indicates Chinese 24-h standard (Grade II) for PM10 (150 μg/m3). Table S1. Coefficient of correlation between the semen parameters. Table S2. Distribution of air pollutant exposure for study subjects. Table S3. Coefficients from linear regression for PM10 exposure in relation to semen parameters by exposure period (0-90, 0-9, 10-14, 15-69, 70-90 days) prior to semen collection in all participants (n = 1554) expressed as change in the parameter for a 10 μg/m3 increase in exposure. Table S4. Coefficients from linear regression for PM2.5 exposure in relation to semen parameters by exposure period (0-90, 0-9, 10-14, 15-69, 70-90 days) prior to semen collection in normal and abnormal semen parameters groups expressed as change in the parameter for a 10 μg/m3 increase in exposure. Table S5. Coefficients from linear regression for PM10 exposure in relation to semen parameters by exposure period (0-90, 0-9, 10-14, 15-69, 70-90 days) prior to semen collection in normal and abn [file 12940_2022_831_MOESM1_ESM.docx]

**Supplemental files**

**Detailed information about the NMU-LIFE cohort.**

*Who is on the cohort?*

Pregnant women that went for registration at the hospital were identified as potential candidates for the study. Maternity care doctors determined the eligible individuals. Exclusion criteria included maternal age < 20 or > 45 years, non-permanent residents, and intention of delivering in other cities. After learning about the study in detail, the women that agreed to participate would represent herself and her family members to sign the informed consent, in other words the whole family was recruited.

*What has been measured?*

Data regarding sociodemographic characteristics, behavioral risk factors for the study participants, both men and women, were measured at baseline examination. The menstrual and reproductive history of the women were recorded. Birth outcomes of offspring were also collected. Additionally, biospecimen including semen, blood, and urine were collected. All study investigators who had a medical background were trained and evaluated to be qualified for working on the specific study tasks.

*Sociodemographic characteristics*

Sociodemographic characteristics were collected by face-to-face questionnaire interview and information of age, ethnicity, height, weight, family address, education level (classified as illiteracy, primary school, junior school, high school, secondary school, and college or above), and annual household income (Chinese Yuan) (<30000, 30000–50000, 50000-100000 and ≥ 100000) are included.

*Behavioral risk factors*

Data were collected by face-to-face questionnaire interviews. Smoking status was classified as currently smoking, ever smoking (have quitted smoking currently) and never smoking. Data on smoking starting time, smoking amount, quitting time, and duration were collected. Drinking status was classified into currently drinking, ever drinking (have quitted drinking currently), drinking from time to time (one or two times a week), and often drinking (equal to or greater than three times a week). Tea and coffee consumption (including type, frequency, and volume), exercise status (every day, 3-6 times a week, 1-2 times a week, and never), and sleeping hours were also investigated by face-to-face questionnaire interview.

*Menstrual and reproductive history*

Data on menstruation (age of menarche, menopause status, and menopause age) and reproduction (contraception, pregnancy, and breastfeeding) for women were collected by face-to-face questionnaire interview.

*Birth outcomes of offspring*

Data on birth weight, gestational age, gender, and Apgar scores of offspring were collected from the hospital medical records system.

**
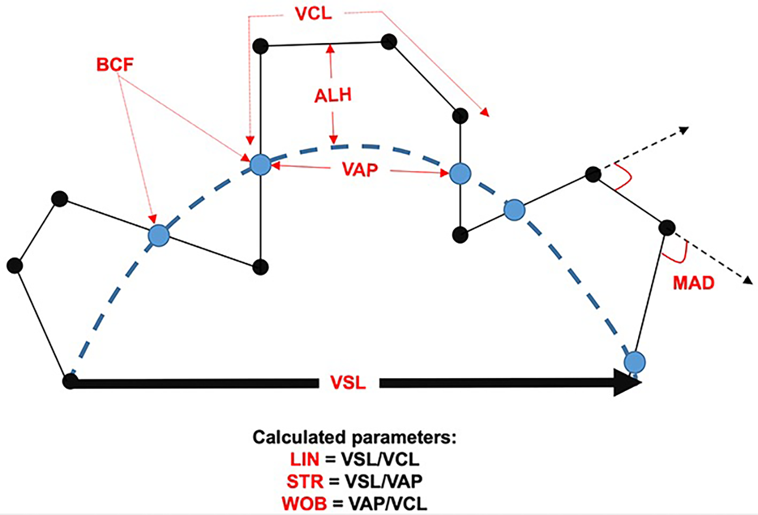
**

**Figure S1. Sperm kinematic parameters measured by computer assisted semen analysis (CASA).**

ALH, amplitude of lateral head displacement; BCF, beat cross frequency; LIN, linearity; MAD, mean angular displacement; STR, straightness; VAP, average path velocity; VCL, curvilinear velocity; VSL, straight line velocity; WOB, curvilinear path wobble.

**
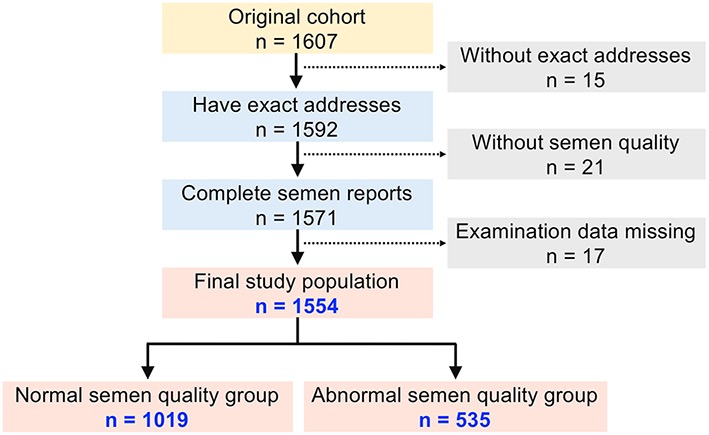
**

**Figure S2. Flowchart of participants in the study.** Normal semen quality group defined by semen volume ≥ 1.5 ml, sperm concentration ≥ 15 × 10^6^/ml, total sperm number ≥ 39 × 10^6^, and total motility ≥ 40%. Abnormal semen quality group defined by at least one abnormal semen parameters (semen volume, sperm concentration, total sperm number or sperm motility).

**
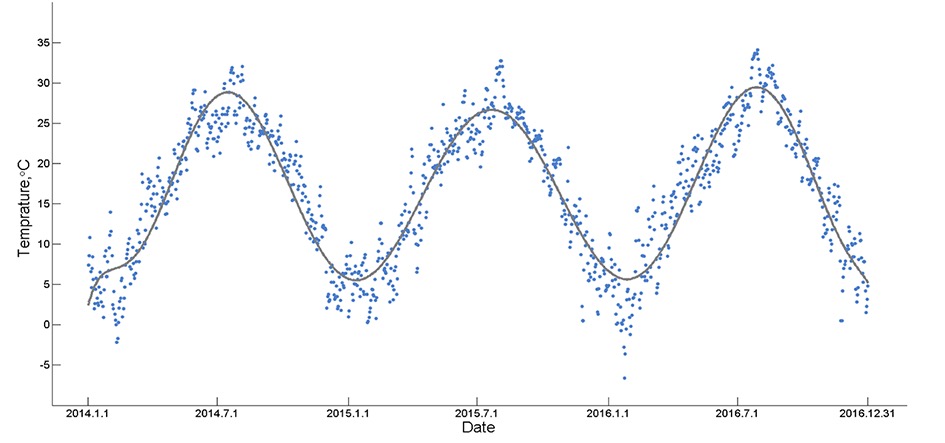
**

**Figure S3. Distribution of daily temperatures in Nanjing between 2014 and 2016.** The points in top and bottom graphs indicate daily temperatures.

**
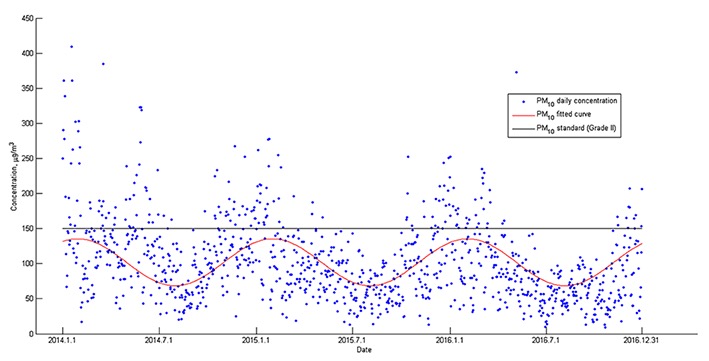
**

**Figure S4. Distribution of daily PM_10_ in Nanjing between 2014 and 2016.** The points in top and bottom graphs indicate daily PM_10_. The straight black line indicates Chinese 24-h standard (Grade II) for PM_10_ (150 μg/m^3^).

**Table S1. Coefficient of correlation between the semen parameters.**

|  | **Semen volume** | **Sperm concentration** | **Total sperm number** | **Total motility** | **Progressive motility** | **VCL** | **VSL** | **VAP** | **BCF** | **ALH** | **LIN** | **STR** | **WOB** | **MAD** |
| --- | --- | --- | --- | --- | --- | --- | --- | --- | --- | --- | --- | --- | --- | --- |
| Semen volume | 1 | 0.09^***^ | 0.68^***^ | -0.04 | -0.05 | -0.14^***^ | -0.12^***^ | -0.14^***^ | 0.05^*^ | -0.08^**^ | 0.01 | -0.01 | 0.02 | -0.04 |
| Sperm concentration |  | 1 | 0.72^***^ | 0.15^***^ | 0.00 | -0.24^***^ | -0.24^***^ | -0.23^***^ | 0.33^***^ | -0.15^***^ | -0.17^***^ | -0.30^***^ | -0.10^***^ | 0.08^***^ |
| Total sperm number |  |  | 1 | 0.05 | -0.06^*^ | -0.25^***^ | -0.25^***^ | -0.26^***^ | 0.27^***^ | -0.15^***^ | -0.12^***^ | -0.22^***^ | -0.06^*^ | 0.02 |
| Total motility |  |  |  | 1 | 0.94^***^ | 0.38^***^ | 0.39^***^ | 0.41^***^ | -0.29^***^ | 0.19^***^ | 0.08^***^ | 0.11^***^ | 0.06^*^ | 0.10^***^ |
| Progressive motility |  |  |  |  | 1 | 0.41^***^ | 0.53^***^ | 0.53^***^ | -0.50^***^ | 0.14^***^ | 0.28^***^ | 0.32^***^ | 0.24^***^ | -0.05 |
| VCL |  |  |  |  |  | 1 | 0.75^***^ | 0.82^***^ | -0.30^***^ | 0.61^***^ | -0.17^***^ | 0.09^***^ | -0.26^***^ | 0.36^***^ |
| VSL |  |  |  |  |  |  | 1 | 0.96^***^ | -0.64^***^ | 0.19^***^ | 0.41^***^ | 0.46^***^ | 0.33^***^ | -0.14^***^ |
| VAP |  |  |  |  |  |  |  | 1 | -0.59^***^ | 0.28^***^ | 0.30^***^ | 0.36^***^ | 0.23^***^ | -0.06^*^ |
| BCF |  |  |  |  |  |  |  |  | 1 | 0.06^***^ | -0.70^***^ | -0.67^***^ | -0.64^***^ | 0.43^***^ |
| ALH |  |  |  |  |  |  |  |  |  | 1 | -0.56^***^ | -0.22^***^ | -0.63^***^ | 0.53^***^ |
| LIN |  |  |  |  |  |  |  |  |  |  | 1 | 0.76^***^ | 0.94^***^ | -0.76^***^ |
| STR |  |  |  |  |  |  |  |  |  |  |  | 1 | 0.58^***^ | -0.41^***^ |
| WOB |  |  |  |  |  |  |  |  |  |  |  |  | 1 | -0.83^***^ |
| MAD |  |  |  |  |  |  |  |  |  |  |  |  |  | 1 |

Note: ALH, amplitude of lateral head displacement; BCF, beat cross frequency; LIN, linearity; MAD, mean angular displacement; PM_2.5_, particulate matter with aerodynamic less than 2.5 μm; SE, standard error; STR, straightness; VAP, average path velocity; VCL, curvilinear velocity; VSL, straight line velocity; WOB, curvilinear path wobble. ^*^*P* < 0.05, ^**^*P* < 0.01, ^***^*P* < 0.001.

**Table S2. Distribution of air pollutant exposure for study subjects.**

| **Exposure period** | **Pollution (μg/m^3^)** | **Mean (SD)** | **Min** | **Max** | **10%** | **25%** | **50%** | **75%** | **90%** |
| --- | --- | --- | --- | --- | --- | --- | --- | --- | --- |
| 0-90 days | PM_2.5_ | 60.80 (13.30) | 28.70 | 92.10 | 41.90 | 52.30 | 60.60 | 70.20 | 79.20 |
|  | PM_10_ | 103.10 (20.50) | 55.60 | 158.80 | 74.90 | 86.50 | 105.60 | 120.60 | 129.30 |

Note: PM_2.5_, particulate matter with aerodynamic less than 2.5 μm; PM_10_, particulate matter with aerodynamic less than 10 μm; SD, standard deviation.

**Table S3. Coefficients from linear regression for PM_10_ exposure in relation to semen parameters by exposure period (0-90, 0-9, 10-14, 15-69, 70-90 days) prior to semen collection in all participants (n = 1,554)** **expressed as change in the parameter for a 10 μg/m^3^ increase in exposure.**

| **Semen parameter^a^** | **0-90 days** | | **0-9 days** | | **10-14 days** | | **15-69 days** | | **70-90 days** | |
| --- | --- | --- | --- | --- | --- | --- | --- | --- | --- | --- |
|  | **Coefficient**  **(×10; 95% CI)** | ***P*^b^** | **Coefficient**  **(×10; 95% CI)** | ***P*^b, c^** | **Coefficient**  **(×10; 95% CI)** | ***P*^b, d^** | **Coefficient**  **(×10; 95% CI)** | ***P*^b, e^** | **Coefficient**  **(×10; 95% CI)** | ***P*^b, f^** |
| Semen volume (ml) | -0.007 (-0.026, -0.011) | 0.760 | -0.006 (-0.016, 0.003) | 0.693 | -0.002 (-0.009, 0.006) | 0.926 | -0.009 (-0.024, -0.007) | 0.892 | 0.011 (-0.0004, 0.022) | 0.077 |
| Concentration (10^6^/ml) | -0.043 (-1.377, 1.290) | 0.949 | -0.286 (-0.942, 0.370) | 0.693 | -0.463 (-0.993, 0.068) | 0.408 | 0.707 (-0.409, 1.824) | 0.892 | -0.460 (-1.227, 0.307) | 0.279 |
| Total sperm number (10^6^) | -0.002 (-0.037, 0.034) | 0.949 | -0.012 (-0.029, 0.006) | 0.693 | -0.012 (-0.026, 0.002) | 0.408 | -0.010 (-0.019, 0.040) | 0.892 | -0.002 (-0.022, 0.018) | 0.844 |
| Total motility (%) | -0.871 (-1.674, -0.068) | 0.117 | -0.249 (-0.643, 0.144) | 0.693 | -0.165 (-0.483, 0.153) | 0.891 | -0.023 (-0.649, 0.696) | 0.957 | -0.407 (-0.869, 0.055) | 0.118 |
| Progressive motility (%) | -0.573 (-1.275, 0.129) | 0.255 | -0.146 (-0.491, 0.199) | 0.693 | -0.097 (-0.375, 0.182) | 0.891 | 0.198 (-0.391, 0.787) | 0.892 | -0.471 (-0.876, -0.067) | 0.077 |
| VCL (μm/s) | **-0.627 (-0.997, -0.262)** | **0.007** | -0.088 (-0.218, 0.093) | 0.693 | -0.028 (-0.174, 0.117) | 0.926 | -0.154 (-0.461, 0.153) | 0.892 | -0.208 (-0.419, -0.002) | 0.081 |
| VSL (μm/s) | -0.012 (-0.021, -0.003) | 0.052 | -0.001 (-0.006, 0.004) | 0.693 | -0.001 (-0.005, 0.002) | 0.891 | -0.002 (-0.010, 0.006) | 0.925 | **-0.009 (-0.014, -0.004)** | **0.005** |
| VAP (μm/s) | **-0.424 (-0.676, -0.172)** | **0.007** | -0.070 (-0.195, 0.055) | 0.693 | -0.018 (-0.118, 0.083) | 0.926 | -0.084 (-0.296, 0.128) | 0.892 | **-0.259 (-0.403, -0.115)** | **0.005** |
| BCF (Hz) | -0.002 (-0.007, 0.004) | 0.782 | -0.001 (-0.004, 0.002) | 0.693 | -0.001 (-0.003, 0.001) | 0.891 | -0.003 (-0.008, 0.002) | 0.892 | 0.004 (0.001, 0.007) | 0.056 |
| ALH (μm/s) | -0.012 (-0.025, 0.001) | 0.181 | 0.001 (-0.005, 0.007) | 0.775 | 0.0001 (-0.005, 0.005) | 0.977 | 0.001 (-0.011, 0.010) | 0.957 | -0.004 (-0.011, 0.003) | 0.313 |
| LIN (%) | 0.062 (-0.252, 0.377) | 0.949 | 0.051 (-0.104, 0.207) | 0.693 | 0.052(-0.074, 0.178) | 0.891 | 0.047 (-0.217, 0.311) | 0.925 | -0.200 (-0.380, -0.020) | 0.077 |
| STR (%) | 0.001 (-0.001, 0.003) | 0.733 | 0.001 (-0.001, 0.002) | 0.693 | 0.001 (0.0001, 0.002) | 0.408 | 0.001 (-0.001, 0.002) | 0.892 | -0.001 (-0.002, -0.0001) | 0.077 |
| WOB (%) | 0.022 (-0.246, 0.290) | 0.949 | 0.031 (-0.101, 0.164) | 0.693 | 0.002 (-0.106, 0.109) | 0.977 | 0.006 (-0.219, 0.232) | 0.957 | -0.158 (-0.311, -0.004) | 0.077 |
| MAD (°) | 0.001 (-0.007, 0.010) | 0.949 | -0.001 (-0.005, 0.003) | 0.693 | -0.0003 (-0.004, 0.003) | 0.977 | 0.001 (-0.006, 0.008) | 0.925 | 0.003 (-0.001, 0.008) | 0.226 |

^a^ALH, amplitude of lateral head displacement; BCF, beat cross frequency; LIN, linearity; MAD, mean angular displacement; PM_10_, particulate matter with aerodynamic less than 10 μm; CI, confidence interval; STR, straightness; VAP, average path velocity; VCL, curvilinear velocity; VSL, straight line velocity; WOB, curvilinear path wobble.

^b^Results were adjusted for age, BMI, ethnicity, education, smoking status, drinking status, family income, abstinence period, season, and temperature. *P* value for adjusting FDR using the Benjamini & Hochberg procedure.

^c^Ambient particulate matter exposure of 10-14 days, 15-69 days, 70-90 days were also adjusted.

^d^Ambient particulate matter exposure of 0-9 days, 15-69 days, 79-90 days were also adjusted.

^e^Ambient particulate matter exposure of 0-9 days, 10-14 days, 70-90 days was also adjusted.

^f^Ambient particulate matter exposure of 0-9 days, 10-14 days, 15-69 days was also adjusted.

**Table S4. Coefficients from linear regression for PM_2.5_ exposure in relation to semen parameters by exposure period (0-90, 0-9, 10-14, 15-69, 70-90 days) prior to semen collection in normal and abnormal semen parameters groups expressed as change in the parameter for a 10 μg/m^3^ increase in exposure.**

| **Semen parameter^a^** | **0-90 days** | | **0-9 days** | | **10-14 days** | | **15-69 days** | | **70-90 days** | |
| --- | --- | --- | --- | --- | --- | --- | --- | --- | --- | --- |
|  | **Coefficient**  **(×10; 95% CI)** | ***P*^b^** | **Coefficient**  **(×10; 95% CI)** | ***P*^b, c^** | **Coefficient**  **(×10; 95% CI)** | ***P*^b, d^** | **Coefficient**  **(×10; 95% CI)** | ***P*^b, e^** | **Coefficient**  **(×10; 95% CI)** | ***P*^b, f^** |
| Normal semen parameters group (n = 1,019)^g^ | | | | | | | | | | |
| Semen volume (ml) | -0.001 (-0.021, 0.018) | 0.968 | -0.003(-0.015, 0.010) | 0.841 | 0.005 (-0.005, 0.015) | 0.862 | -0.0001 (-0.019, 0.018) | 0.991 | -0.003 (-0.017, 0.011) | 0.796 |
| Sperm concentration (10^6^/ml) | 0.320 (-1.486, 2.127) | 0.968 | -0.282 (-1.430, 0.867) | 0.841 | -0.119 (-1.062, 0.825) | 0.890 | 1.221 (-0.501, 2.923) | 0.331 | 0.202 (-1.523, 1.120) | 0.824 |
| Total sperm number (10^6^) | -0.005 (-0.029, 0.039) | 0.968 | -0.008 (-0.030, 0.013) | 0.841 | 0.005 (-0.013, 0.023) | 0.862 | 0.019 (-0.014, 0.051) | 0.459 | 0.005 (-0.030, 0.020) | 0.796 |
| Total motility (%) | **-1.444 (-2.267, -0.621)** | **0.002** | -0.391 (-0.915, 0.133) | 0.501 | -0.128 (-0.302, 0.558) | 0.862 | -0.564 (-1.347, 0.219) | 0.331 | -0.647 (-1.253, -0.041) | 0.116 |
| Progressive motility (%) | **-1.370 (-2.146, -0.595)** | **0.002** | -0.309 (-0.805, 0.186) | 0.619 | 0.184 (-0.222, 0.590) | 0.862 | -0.583 (-1.323, 0.157) | 0.331 | -0.790 (-1.358, -0.222) | 0.090 |
| VCL (μm/s) | **-1.114 (-1.593, -0.635)** | **< 0.001** | -0.283 (-0.588, 0.022) | 0.321 | -0.064 (-0.186, 0.314) | 0.862 | **-0.917 (-1.372, -0.462)** | **0.001** | -0.155 (-0.504, -0.194) | 0.538 |
| VSL (μm/s) | **-0.023 (-0.034, -0.011)** | **< 0.001** | -0.009 (-0.016, -0.002) | 0.096 | -0.001 (-0.005, 0.006) | 0.890 | **-0.018 (-0.028, -0.007)** | **0.004** | -0.008 (-0.016, -0.0001) | 0.116 |
| VAP (μm/s) | **-0.733 (-1.067, -0.400)** | **< 0.001** | -0.279 (-0.492, -0.067) | 0.096 | -0.047 (-0.127, 0.222) | 0.862 | **-0.594 (-0.910, -0.278)** | **0.002** | -0.230 (-0.468, -0.008) | 0.116 |
| BCF (Hz) | 0.001 (-0.006, 0.009) | 0.968 | 0.0001 (-0.005, 0.005) | 0.972 | -0.001 (-0.005, 0.003) | 0.862 | 0.003 (-0.004, 0.009) | 0.748 | 0.004 (0.001, 0.009) | 0.203 |
| ALH (μm/s) | **-0.024 (-0.041, -0.008)** | **0.008** | -0.0004 (-0.011, 0.010) | 0.972 | 0.004 (-0.005, 0.012) | 0.862 | -0.019 (-0.034, -0.003) | 0.061 | -0.001 (-0.013, 0.011) | 0.905 |
| LIN (%) | -0.0002 (-0.445, 0.445) | 0.999 | -0.072 (-0.358, 0.212) | 0.841 | 0.037 (-0.197, 0.270) | 0.890 | 0.034 (-0.389, 0.458) | 0.957 | -0.342 (-0.665, -0.019) | 0.116 |
| STR (%) | -0.001 (-0.003, 0.002) | 0.968 | -0.001 (-0.002, 0.001) | 0.841 | 0.001 (-0.001, 0.002) | 0.862 | -0.001 (-0.003, 0.002) | 0.908 | -0.002 (-0.003, -0.0003) | 0.179 |
| WOB (%) | 0.033 (-0.343, 0.408) | 0.968 | -0.058 (-0.297, 0.181) | 0.841 | -0.002 (-0.198, 0.195) | 0.988 | 0.057 (-0.301, 0.414) | 0.957 | -0.281 (-0.554, -0.009) | 0.116 |
| MAD (°) | 0.001 (-0.007, 0.010) | 0.968 | 0.0002 (-0.005, 0.006) | 0.972 | 0.002 (-0.003, 0.006) | 0.862 | 0.001 (-0.009, 0.007) | 0.957 | 0.006 (0.0001, 0.012) | 0.116 |
| Abnormal semen parameters group (n = 535)^h^ | | | | | | | | | | |
| Semen volume (ml) | 0.005 (-0.037, 0.048) | 0.963 | -0.004(-0.033, 0.024) | 0.994 | 0.002 (-0.021, 0.025) | 0.948 | -0.002 (-0.048, 0.044) | 0.923 | 0.023 (-0.009, 0.056) | 0.452 |
| Sperm concentration (10^6^/ml) | 0.8331 (-1.785, 3.451) | 0.828 | 0.111 (-1.637, 1.859) | 0.994 | -0.300 (-1.729, 1.129) | 0.948 | 1.722 (-1.130, 4.574) | 0.826 | -0.782 (-2.821，1.257) | 0.778 |
| Total sperm number (10^6^) | 0.027 (-0.054, 0.108) | 0.828 | 0.001 (-0.053, 0.055) | 0.994 | -0.007 (-0.051, 0.037) | 0.948 | 0.041 (-0.047, 0.129) | 0.879 | 0.0002 (-0.063, 0.063) | 0.955 |
| Total motility (%) | -1.618 (-2.842, -0.052) | 0.068 | -0.392 (-1.341, 0.557) | 0.962 | -0.192 (-0.918, 0.584) | 0.948 | -1.431 (-2.980, 0.118) | 0.584 | -0.178 (-1.285, 0.929) | 0.810 |
| Progressive motility (%) | -0.014 (-0.035, -0.007) | 0.068 | -0.250 (-1.069, 0.570) | 0.962 | -0.067 (-0.736, 0.603) | 0.948 | -1.181 (-2.517, 0.157) | 0.584 | -0.242 (-1.196, 0.713) | 0.788 |
| VCL (μm/s) | -0.460(-0.968, 0.048) | 0.265 | -0.191 (-0.698, 0.317) | 0.962 | -0.008 (-0.423, 0.406) | 0.969 | -0.202 (-1.030, 0.626) | 0.923 | -0.462 (-1.052, 0.127) | 0.434 |
| VSL (μm/s) | 0.005 (-0.007, 0.016) | 0.574 | -0.0001 (-0.014, 0.014) | 0.994 | 0.001 (-0.010, 0.013) | 0.948 | 0.004 (-0.027, 0.019) | 0.923 | -0.021 (-0.037, -0.004) | 0.086 |
| VAP (μm/s) | -0.016 (-0.043, 0.012) | 0.265 | -0.050 (-0.389, 0.289) | 0.994 | 0.047 (-0.229, 0.324) | 0.948 | 0.090 (-0.642 0.462) | 0.602 | **-0.589 (-0.979, -0.199)** | **0.044** |
| BCF (Hz) | 0.038(-0.571, 0.6472 | 0.828 | -0.001 (-0.008, 0.007) | 0.994 | -0.002 (-0.008, 0.004) | 0.948 | -0.001 (-0.011, 0.014) | 0.923 | 0.009 (0.0002, 0.018) | 0.207 |
| ALH (μm/s) | 0.0004(-0.004, 0.005) | 0.642 | 0.008 (-0.010, 0.027) | 0.962 | -0.007 (-0.022, 0.008) | 0.948 | -0.006 (-0.037, 0.024) | 0.923 | -0.010 (-0.032, 0.012) | 0.744 |
| LIN (%) | 0.012 (-0.513, 0.538) | 0.963 | 0.153 (-0.253, 0.559) | 0.962 | 0.216 (-0.116, 0.547) | 0.948 | -0.298 (-0.959, 0.363) | 0.879 | -0.130 (-0.599, 0.339) | 0.788 |
| STR (%) | 0.005 (-0.026, 0.016) | 0.963 | 0.001 (-0.002, 0.004) | 0.962 | 0.002 (-0.001, 0.004) | 0.948 | 0.0003 (-0.005, 0.005) | 0.923 | -0.002 (-0.006, 0.0001) | 0.559 |
| WOB (%) | -0.028 (-0.556, 0.500) | 0.963 | 0.120 (-0.231, 0.471) | 0.962 | 0.120 (-0.166, 0.407) | 0.948 | -0.174 (-0.746, 0.398) | 0.923 | -0.139 (-0.546, 0.267) | 0.778 |
| MAD (°) | -0.003 (-0.025, 0.019) | 0.896 | -0.008 (-0.022, 0.007) | 0.962 | -0.008 (-0.020, 0.003) | 0.948 | -0.006 (-0.017, 0.030) | 0.923 | 0.004 (-0.013, 0.020) | 0.790 |

^a^ALH, amplitude of lateral head displacement; BCF, beat cross frequency; LIN, linearity; MAD, mean angular displacement; PM_2.5_, particulate matter with aerodynamic less than 2.5 μm; CI, confidence interval; STR, straightness; VAP, average path velocity; VCL, curvilinear velocity; VSL, straight line velocity; WOB, curvilinear path wobble.

^b^ Results were adjusted for age, BMI, ethnicity, education, smoking status, drinking status, family income, abstinence period, season and temperature. *P* value for adjusting FDR using the Benjamini & Hochberg procedure.

^c^Ambient particulate matter exposure of 10-14 days, 15-69 days, 70-90 days were also adjusted.

^d^Ambient particulate matter exposure of 0-9 days, 15-69 days, 79-90 days were also adjusted.

^e^Ambient particulate matter exposure of 0-9 days, 10-14 days, 70-90 days was also adjusted.

^f^Ambient particulate matter exposure of 0-9 days, 10-14 days, 15-69 days was also adjusted.

^g^Group defined by semen volume ≥ 1.5 ml, sperm concentration ≥ 15 × 10^6^/ml, total sperm number ≥ 39 × 10^6^, and total motility ≥ 40%.

^h^Group defined by at least one abnormal semen parameters (semen volume, sperm concentration, total sperm number or total motility).

**Table S5. Coefficients from linear regression for PM_10_ exposure in relation to semen parameters by exposure period (0-90, 0-9, 10-14, 15-69, 70-90 days) prior to semen collection in normal and abnormal semen parameters groups** **expressed as change in the parameter for a 10 μg/m^3^ increase in exposure.**

| **Semen parameter^a^** | **0-90 days** | | **0-9 days** | | **10-14 days** | | **15-69 days** | | **70-90 days** | |
| --- | --- | --- | --- | --- | --- | --- | --- | --- | --- | --- |
|  | **Coefficient**  **(×10; 95% CI)** | ***P* ^b^** | **Coefficient**  **(×10; 95% CI)** | ***P* ^b, c^** | **Coefficient**  **(×10; 95% CI)** | ***P* ^b, d^** | **Coefficient**  **(×10; 95% CI)** | ***P* ^b, e^** | **Coefficient**  **(×10; 95% CI)** | ***P* ^b, f^** |
| Normal semen parameters group (n = 1,019)^g^ | | | | | | | | | | |
| Semen volume (ml) | 0.007 (-0.010, 0.024) | 0.700 | -0.002 (-0.010, 0.006) | 0.841 | 0.002 (-0.005, 0.008) | 0.870 | -0.001 (-0.015, 0.013) | 0.947 | 0.006 (-0.004, 0.016) | 0.274 |
| Sperm concentration (10^6^/ml) | -0.221 (-1.778, 1.337) | 0.911 | -0.202 (-0.954, 0.550) | 0.841 | -0.366 (-0.983, 0.251) | 0.870 | 0.393 (-0.880, 1.665) | 0.940 | -0.420 (-1.323, 0.484) | 0.422 |
| Total sperm number (10^6^) | 0.007 (-0.023, 0.036) | 0.843 | -0.006 (-0.021, 0.008) | 0.841 | -0.004 (-0.015, 0.008) | 0.870 | 0.003 (-0.021, 0.027) | 0.940 | -0.001 (-0.018, 0.016) | 0.913 |
| Total motility (%) | -0.537 (-1.250, 0.176) | 0.405 | -0.332 (-0.674, 0.010) | 0.267 | -0.051 (-0.332, 0.229) | 0.870 | 0.161 (-0.420, 0.743) | 0.940 | -0.436 (-0.849, -0.023) | 0.067 |
| Progressive motility (%) | -0.320 (-0.992, 0.352) | 0.700 | -0.261 (-0.585, 0.064) | 0.322 | 0.026 (-0.239, 0.291) | 0.870 | 0.287 (-0.263, 0.838) | 0.940 | -0.554 (-0.943, -0.166) | 0.057 |
| VCL (μm/s) | **-0.750 (-1.165, -0.336)** | **0.003** | -0.217 (-0.418, -0.016) | 0.243 | 0.014 (-0.151, 0.178) | 0.870 | -0.265 (-0.606, 0.075) | 0.924 | -0.156 (-0.397, 0.085) | 0.274 |
| VSL (μm/s) | **-0.015 (-0.025, -0.005)** | **0.010** | -0.004 (-0.009, 0.001) | 0.322 | -0.001 (-0.005, 0.003) | 0.870 | -0.005 (-0.012, 0.003) | 0.940 | -0.007 (-0.013, -0.002) | 0.057 |
| VAP (μm/s) | **-0.515 (-0.803, -0.227)** | **0.003** | -0.154 (-0.294, -0.014) | 0.243 | -0.015 (-0.130, 0.099) | 0.870 | -0.182 (-0.418, 0.055) | 0.924 | -0.201 (-0.366, -0.036) | 0.057 |
| BCF (Hz) | -0.003 (-0.009, 0.004) | 0.700 | -0.0004 (-0.003, 0.003) | 0.842 | -0.0005 (-0.003, 0.002) | 0.870 | -0.002 (-0.007, 0.003) | 0.940 | 0.003 (-0.0002, 0.007) | 0.102 |
| ALH (μm/s) | -0.010 (-0.025, 0.004) | 0.405 | -0.002 (-0.009, 0.004) | 0.841 | 0.001 (-0.004, 0.007) | 0.870 | -0.002 (-0.013, 0.010) | 0.940 | 0.001 (-0.007, 0.009) | 0.881 |
| LIN (%) | 0.023 (-0.361, 0.407) | 0.976 | 0.029 (-0.156, 0.215) | 0.842 | 0.017 (-0.135, 0.170) | 0.870 | 0.070 (-0.245, 0.385) | 0.940 | -0.274 (-0.494, -0.053) | 0.057 |
| STR (%) | 0.001 (-0.002, 0.003) | 0.824 | 0.0001 (-0.001, 0.001) | 0.925 | 0.001 (-0.0004, 0.001) | 0.870 | 0.001 (-0.001, 0.003) | 0.940 | -0.001 (-0.003, 0.0001) | 0.067 |
| WOB (%) | 0.003 (-0.320, 0.327) | 0.983 | 0.025 (-0.132, 0.182) | 0.842 | -0.012 (-0.141, 0.116) | 0.870 | 0.009 (-0.257, 0.275) | 0.947 | -0.215 (-0.401, -0.028) | 0.057 |
| MAD (°) | 0.002 (-0.005, 0.010) | 0.799 | -0.001 (-0.005, 0.002) | 0.841 | 0.002 (-0.001, 0.005) | 0.870 | 0.001 (-0.005, 0.007) | 0.940 | 0.005 (0.001, 0.009) | 0.057 |
| Abnormal semen parameters group (n = 535)^h^ | | | | | | | | | | |
| Semen volume (ml) | 0.033 (-0.070, 0.005) | 0.863 | -0.012 (-0.032, 0.008) | 0.734 | 0.0002 (-0.015, 0.016) | 0.998 | -0.024 (-0.058, 0.010) | 0.983 | 0.019 (-0.003, 0.041) | 0.410 |
| Sperm concentration (10^6^/ml) | 0.510 (-1.829, 2.849) | 0.863 | -0.345 (-1.578, 0.887) | 0.734 | -0.344 (-1.306, 0.619) | 0.913 | 1.151 (-0.950, 3.251) | 0.983 | -0.497 (-1.860, 0.866) | 0.842 |
| Total sperm number (10^6^) | -0.010 (-0.082, 0.062) | 0.863 | -0.018 (-0.057, 0.020) | 0.734 | -0.009 (-0.038, 0.021) | 0.913 | 0.017 (-0.048, 0.082) | 0.983 | -0.002 (-0.044, 0.040) | 0.962 |
| Total motility (%) | -0.923 (-2.198, 0.352) | 0.863 | -0.165 (-0.836, 0.506) | 0.734 | -0.097 (-0.621, 0.427) | 0.913 | -0.388 (-1.533, 0.757) | 0.983 | 0.031 (-0.711, 0.772) | 0.962 |
| Progressive motility (%) | -0.581 (-1.681, 0.518) | 0.863 | 0.049 (-0.532, 0.630) | 0.874 | -0.101 (-0.553, 0.352) | 0.913 | -0.103 (-1.092, 0.886) | 0.983 | -0.016 (-0.657, 0.626) | 0.962 |
| VCL (μm/s) | -0.287 (-0.967, 0.394) | 0.863 | 0.131 (-0.226, 0.489) | 0.734 | -0.083 (-0.362, 0.196) | 0.913 | 0.084 (-0.524, 0.693) | 0.983 | -0.252 (-0.646, 0.143) | 0.494 |
| VSL (μm/s) | -0.004 (-0.022, 0.015) | 0.863 | 0.004 (-0.006, 0.014) | 0.734 | -0.001 (-0.009, 0.007) | 0.939 | 0.003 (-0.014, 0.020) | 0.983 | -0.011 (-0.022, 0.00003) | 0.383 |
| VAP (μm/s) | -0.175 (0.630, 0.280) | 0.863 | 0.075 (-0.165, 0.315) | 0.734 | -0.0002 (-0.187, 0.186) | 0.998 | 0.113 (-0.294, 0.520) | 0.983 | -0.319 (-0.582, -0.055) | 0.249 |
| BCF (Hz) | -0.001 (-0.012, 0.009) | 0.863 | -0.002 (-0.007, 0.004) | 0.734 | -0.002 (-0.006, 0.003) | 0.913 | -0.004 (-0.014, 0.005) | 0.983 | 0.005 (-0.001, 0.011) | 0.410 |
| ALH (μm/s) | -0.012 (-0.036, 0.013) | 0.863 | 0.006 (-0.007, 0.019) | 0.734 | -0.003 (-0.013, 0.007) | 0.913 | -0.0002 (-0.022, 0.022) | 0.983 | -0.009 (-0.024, 0.005) | 0.494 |
| LIN (%) | 0.131 (-0.413, 0.675) | 0.863 | 0.127 (-0.158, 0.413) | 0.734 | 0.148 (-0.074, 0.371) | 0.891 | -0.051 (-0.535, 0.434) | 0.983 | -0.062 (-0.375, 0.251) | 0.962 |
| STR (%) | 0.002 (-0.003, 0.006) | 0.863 | 0.001 (-0.001, 0.004) | 0.734 | 0.001 (-0.0002, 0.003) | 0.891 | -0.0001 (-0.004, 0.004) | 0.983 | -0.001 (-0.003, 0.002) | 0.842 |
| WOB (%) | 0.048 (-0.421, 0.518) | 0.863 | 0.079 (-0.168, 0.327) | 0.734 | 0.048 (-0.145, 0.241) | 0.913 | -0.012 (-0.433, 0.408) | 0.983 | -0.062 (-0.334, 0.210) | 0.962 |
| MAD (°) | -0.002 (-0.021, 0.017) | 0.863 | -0.001 (-0.011, 0.009) | 0.874 | -0.005 (-0.013, 0.003) | 0.891 | 0.006 (-0.012, 0.023) | 0.983 | -0.001 (-0.012, 0.010) | 0.962 |

^a^ALH, amplitude of lateral head displacement; BCF, beat cross frequency; LIN, linearity; MAD, mean angular displacement; PM_10_, particulate matter with aerodynamic less than 10 μm; CI, confidence interval; STR, straightness; VAP, average path velocity; VCL, curvilinear velocity; VSL, straight line velocity; WOB, curvilinear path wobble.

^b^ Results were adjusted for age, BMI, ethnicity, education, smoking status, drinking status, family income, abstinence period, season and temperature. *P* value for adjusting FDR using the Benjamini & Hochberg procedure.

^c^Ambient particulate matter exposure of 10-14 days, 15-69 days, 70-90 days were also adjusted.

^d^Ambient particulate matter exposure of 0-9 days, 15-69 days, 79-90 days were also adjusted.

^e^Ambient particulate matter exposure of 0-9 days, 10-14 days, 70-90 days was also adjusted.

^f^Ambient particulate matter exposure of 0-9 days, 10-14 days, 15-69 days was also adjusted.

^g^Group defined by semen volume ≥ 1.5 ml, sperm concentration ≥ 15 × 10^6^/ml, total sperm number ≥ 39 × 10^6^, and total motility ≥ 40%.

^h^Group defined by at least one abnormal semen parameters (semen volume, sperm concentration, total sperm number or total motility).

**Table S6. Coefficients (95% CIs) from linear regression of PM_2.5_ exposure during 0-90 days before semen collection in relation to sperm parameters in normal semen parameters group expressed as change in the parameter for a 10 μg/m^3^ increase in exposure.**

| **Semen parameter^a^** | **Quintile of PM_2.5_ exposure (range)** | | | | | ***P* trend^b^** |
| --- | --- | --- | --- | --- | --- | --- |
|  | **Q1 (28.7-50.0)** | **Q2 (50.1-57.4)** | **Q3 (57.5-64.7)** | **Q4 (64.8-73.8)** | **Q5 (73.9-92.1)** |  |
| Semen volume (ml) | 0 (reference) | -0.01 (-0.08, 0.05) | -0.05 (-0.13, 0.03) | -0.09 (-0.17, -0.01) | -0.04 (-0.12, 0.04) | 0.474 |
| Sperm concentration (10^6^/ml) | 0 (reference) | 3.78 (-2.46, 10.02) | 3.38 (-3.67, 10.44) | -0.68 (-7.82, 6.46) | 0.91 (-6.17, 7.98) | 0.889 |
| Total sperm number (10^6^) | 0 (reference) | 0.06 (-0.06, 0.18) | 0.02 (-0.12, 0.15) | -0.08 (-0.22, 0.05) | -0.02 (-0.15, 0.12) | 0.752 |
| Total motility (%) | 0 (reference) | -3.19 (-6.03, -0.34) | -3.52 (-6.74, -0.30) | -2.91 (-6.17, 0.34) | -5.56 (-8.79, -2.33) | 0.006 |
| Progressive motility (%) | 0 (reference) | -2.90 (-5.58, -0.23) | -2.38 (-5.41, 0.64) | -1.18 (-4.25, 1.88) | -5.31 (-8.35, -2.28) | 0.006 |
| VCL (μm/s) | 0 (reference) | 0.67 (-0.98, 2.33) | -0.57 (-2.44, 1.30) | -0.12 (-2.01, 1.78) | -3.16 (-5.03, -1.28) | 0.002 |
| VSL (μm/s) | 0 (reference) | 0.002 (-0.04, 0.04) | -0.01 (-0.05, 0.03) | 0.02 (-0.02, 0.06) | -0.07 (-0.12, -0.03) | 0.003 |
| VAP (μm/s) | 0 (reference) | 0.14 (-1.01, 1.29) | -0.27 (-1.57, 1.02) | 0.43 (-0.88, 1.75) | -2.31 (-3.61, -1.01) | 0.002 |
| BCF (Hz) | 0 (reference) | 0.004 (-0.02, 0.03) | -0.02 (-0.04, 0.01) | -0.04 (-0.07, -0.01) | 0.01 (-0.02, 0.04) | 0.889 |
| ALH (μm/s) | 0 (reference) | 0.01 (-0.04, 0.07) | -0.02 (-0.09, 0.04) | -0.01 (-0.08, 0.04) | -0.06 (-0.13, 0.001) | 0.065 |
| LIN (%) | 0 (reference) | -0.88 (-2.42, 0.65) | 0.50 (-1.23, 2.24) | 0.95 (-0.80, 2.71) | -0.60 (-2.34, 1.14) | 0.889 |
| STR (%) | 0 (reference) | -0.003 (-0.01, 0.01) | 0.003 (-0.01, 0.01) | 0.01 (-0.004, 0.02) | -0.004 (-0.01, 0.01) | 0.780 |
| WOB (%) | 0 (reference) | -0.64 (-1.94, 0.65) | 0.35 (-1.11, 1.82) | 0.81 (-0.67, 2.29) | -0.35 (-1.82, 1.12) | 0.905 |
| MAD (°) | 0 (reference) | 0.02 (-0.01, 0.05) | 0.001 (-0.03, 0.03) | -0.01 (-0.04, 0.02) | 0.02 (-0.01, 0.05) | 0.764 |

^a^ALH, amplitude of lateral head displacement; BCF, beat cross frequency; CI, confidence interval; LIN, linearity; MAD, mean angular displacement; PM_2.5_, particulate matter with aerodynamic less than 2.5 μm; STR, straightness; VAP, average path velocity; VCL, curvilinear velocity; VSL, straight line velocity; WOB, curvilinear path wobble.

^b^*P* trend value for adjusting FDR using the Benjamini & Hochberg procedure.

The coefficients and 95% CIs were estimated using linear model, adjusting for age, BMI, ethnic, education, family income, smoking status, drinking status, abstinence period, season and temperature. Natural log transformation was applied for some sperm parameters.

**Table S7. Coefficients (95% CIs) from linear regression of PM_2.5_ exposure during 0-90 days before semen collection in relation to sperm parameters in abnormal semen parameters group expressed as change in the parameter for a 10 μg/m^3^ increase in exposure.**

| **Semen parameter^a^** | **Quintile of PM_2.5_ exposure (range)** | | | | | ***P* trend^b^** |
| --- | --- | --- | --- | --- | --- | --- |
|  | **Q1 (30.6-49.5)** | **Q2 (49.6-56.3)** | **Q3 (56.4-65.1)** | **Q4 (65.2-74.2)** | **Q5 (74.3-86.3)** |  |
| Semen volume (ml) | 0 (reference) | 0.11 (-0.05, 0.27) | -0.12 (-0.29, 0.05) | -0.02 (-0.20, 0.16) | 0.01 (-0.17, 0.19) | 0.771 |
| Sperm concentration (10^6^/ml) | 0 (reference) | 1.22 (-7.81, 10.24) | 6.94 (-2.89, 16.78) | 4.83 (-5.48, 15.14) | 1.70 (-8.56, 11.97) | 0.668 |
| Total sperm number (10^6^) | 0 (reference) | 0.19 (-0.10, 0.47) | 0.19 (-0.12, 0.50) | 0.18 (-0.15, 0.50) | 0.04 (-0.29, 0.36) | 0.951 |
| Total motility (%) | 0 (reference) | -9.41 (-14.52, -4.31) | -4.12 (-9.69, 1.43) | -9.26 (-15.09, -3.43) | -7.35 (-13.16, -1.55) | 0.546 |
| Progressive motility (%) | 0 (reference) | -7.18 (-11.57, -2.80) | -3.79 (-8.56, 0.99) | -6.75 (-11.76, -1.74) | -6.19 (-11.17, -1.20) | 0.546 |
| VCL (μm/s) | 0 (reference) | -1.92 (-4.59, 0.75) | -1.80 (-4.71, 1.11) | -2.22 (-5.27, 0.83) | -2.77 (-5.80, 0.27) | 0.546 |
| VSL (μm/s) | 0 (reference) | -0.02 (-0.09, 0.06) | -0.01 (-0.09, 0.07) | -0.03 (-0.11, 0.05) | -0.06 (-0.14, 0.02) | 0.567 |
| VAP (μm/s) | 0 (reference) | -0.80 (-2.59, 0.98) | -0.46 (-2.41, 1.49) | -1.34 (-3.39, 0.70) | -1.62 (-3.66, 0.41) | 0.546 |
| BCF (Hz) | 0 (reference) | -0.01 (-0.05, 0.03) | -0.01 (-0.06, 0.03) | -0.02(-0.07, 0.02) | 0.02 (-0.03, 0.06) | 0.668 |
| ALH (μm/s) | 0 (reference) | -0.01 (-0.10, 0.09) | 0.03 (-0.07, 0.14) | -0.06 (-0.17, 0.05) | -0.04 (-0.15, 0.07) | 0.567 |
| LIN (%) | 0 (reference) | 0.58 (-1.53, 2.69) | 0.32 (-1.98, 2.62) | 0.76 (-1.65, 3.17) | 0.10 (-2.30, 2.50) | 0.951 |
| STR (%) | 0 (reference) | -0.003 (-0.01, 0.01) | 0.003 (-0.01, 0.01) | 0.01 (-0.004, 0.02) | -0.004 (-0.01, 0.01) | 0.951 |
| WOB (%) | 0 (reference) | 0.32 (-1.50, 2.15) | -0.01 (-2.00, 1.97) | 0.35 (-1.73, 2.43) | 0.05 (-2.02, 2.13) | 0.951 |
| MAD (°) | 0 (reference) | -0.03 (-0.11, 0.04) | -0.01 (-0.09, 0.07) | -0.003 (-0.09, 0.08) | -0.02 (-0.11, 0.06) | 0.951 |

^a^ALH, amplitude of lateral head displacement; BCF, beat cross frequency; CI, confidence interval; LIN, linearity; MAD, mean angular displacement; PM_2.5_, particulate matter with aerodynamic less than 2.5 μm; STR, straightness; VAP, average path velocity; VCL, curvilinear velocity; VSL, straight line velocity; WOB, curvilinear path wobble.

^b^*P* trend value for adjusting FDR using the Benjamini & Hochberg procedure.

**Table S8.** **Coefficients from linear regression for PM_2.5_ exposure in relation to total sperm motility by categories of age, BMI, income, cigarette smoking and alcohol drinking expressed as change in the parameter for a 10 μg/m^3^ increase in exposure.**

| **Subgroups** | **0-90 days PM_2.5_** | |
| --- | --- | --- |
|  | **Coefficient**  **(×10; 95% CI)** | ***P*** |
| **Age, years** | | |
| < 35 (n = 1279) | -2.478 (-3.482, -1.474) | < 0.001 |
| ≥ 35 (n = 275) | -1.558 (-3.841, 0.725) | 0.180 |
| **BMI, kg/m^2^** | | |
| < 24 (n = 668) | -2.308 (-3.680, -0.936) | 0.001 |
| ≥ 24 (n = 886) | -2.167 (-3.389, -0.945) | < 0.001 |
| **Family income** | | |
| < 100,000 yuan (n = 558) | -2.479 (-3.989, -0.970) | 0.001 |
| ≥ 100,000 yuan (n = 996) | -1.872 (-3.025, -0.719) | 0.001 |
| **Cigarette smoking** | | |
| Never (n = 968) | -2.802 (-3.961, -1.642) | < 0.001 |
| Former or current (n = 586) | -1.463 (-2.945, 0.020) | 0.053 |
| **Alcohol drinking** | | |
| Never (n = 862) | -3.639 (-4.944, -2.334) | < 0.001 |
| Former or current (n = 692) | -0.852 (-2.142, 0.438) | 0.195 |

Note: BMI, body mass index; CI, confidence interval; PM_2.5_, particulate matter with aerodynamic less than 2.5 μm.

**Table S9. Characteristics and main results of previous studies examined the association between PM and semen quality.**

| **First author** | **Year** | **Participants' Characteristics** | **N** | **Type of study** | **Pollutants** | **Significant association** |
| --- | --- | --- | --- | --- | --- | --- |
| Selevan SG^1^ | 2000 | Healthy young men | 272 | Prospective cohort | PM_10_ | Decreased morphology |
| Rubes J^2^ | 2005 | Healthy young men from a highly polluted urban area | 36 | Prospective cohort | PM_10_ | No significant association |
| Sokol RZ^3^ | 2006 | Sperm donors | 48 | Retrospective cohort | PM_10_ | No significant association |
| Hammoud A^4^ | 2010 | Samples from patients from an andrology laboratory | 1465 | Retrospective cohort | PM_2.5_ | Decreased motility |
| Hansen C^5^ | 2010 | Presumed fertile men (partners of pregnant women) | 228 | Retrospective cohort | PM_2.5_ | No significant association |
| Zhou N^6^ | 2014 | Health men | 1346 | Cross-sectional cohort | PM_10_ | Decreased concentration, morphology, VCL, and VSL |
| Radwan M^7^ | 2016 | Patients from a fertility clinic who had normal sperm concentration | 327 | Prospective cohort | PM_2.5_, PM_10_ | Decreased morphology for both PM_2.5_ and PM_10_ |
| Santi D^8^ | 2016 | Samples from patients from a reference laboratory in a general hospital | 406 | Retrospective cohort | PM_2.5_, PM_10_ | Positively associated with total sperm number for PM_2.5_  Positively associated with semen volume for PM_10_ |
| Wu L^9^ | 2017 | Patients from a fertility clinic who had normal sperm concentration | 1759 | Retrospective cohort | PM_2.5_, PM_10_ | Decreased sperm concentration and sperm count for both PM_2.5_ and PM_10_ |
| Lao XQ^10^ | 2017 | Men participated in a standard medical examination programme | 6475 | Cross-sectional cohort | PM_2.5_ | Decreased sperm morphology and increased sperm concentration |
| Zhou N^11^ | 2018 | College students | 796 | Prospective cohort | PM_2.5_, PM_10-2.5_, PM_10_ | Decreased sperm morphology and increased sperm progressive motility for PM_10_  Decreased sperm concentration for PM_10-2.5_  No significant association for PM_2.5_ |
| Santi D^12^ | 2018 | Patients undergo seminal examinations | 5131 | Retrospective cohort | PM_2.5_, PM_10_ | Both PM_2.5_ and PM_10_ inversely related to progressive motility |
| Nobles CJ^13^ | 2018 | Men of couples attempting pregnancy | 467 | Prospective cohort | PM_2.5_ | Not associated with semen quality, except for sperm head parameters |
| Huang X^14^ | 2019 | Men attending a fertility center | 1081 | Retrospective cohort | PM_2.5_ | Decreased sperm concentration and total sperm number |

Note: PM_2.5_, particulate matter with aerodynamic less than 2.5 μm; PM_10_, particulate matter with aerodynamic less than 10 μm.

**References**

1. Selevan SG, Borkovec L, Slott VL, et al. Semen quality and reproductive health of young Czech men exposed to seasonal air pollution. Environ Health Perspect. 2000; 108(9): 887-94.

2. Rubes J, Selevan SG, Evenson DP, et al. Episodic air pollution is associated with increased DNA fragmentation in human sperm without other changes in semen quality. Hum Reprod. 2005; 20(10): 2776-83.

3. Sokol RZ, Kraft P, Fowler IM, Mamet R, Kim E, Berhane KT. Exposure to environmental ozone alters semen quality. Environ Health Perspect. 2006; 114(3): 360-5.

4. Hammoud A, Carrell DT, Gibson M, Sanderson M, Parker-Jones K, Peterson CM. Decreased sperm motility is associated with air pollution in Salt Lake City. Fertil Steril. 2010; 93(6): 1875-9.

5. Hansen C, Luben TJ, Sacks JD, et al. The effect of ambient air pollution on sperm quality. Environ Health Perspect. 2010; 118(2): 203-9.

6. Zhou N, Cui Z, Yang S, et al. Air pollution and decreased semen quality: a comparative study of Chongqing urban and rural areas. Environ Pollut. 2014; 187: 145-52.

7. Radwan M, Jurewicz J, Polanska K, et al. Exposure to ambient air pollution--does it affect semen quality and the level of reproductive hormones? Ann Hum Biol. 2016; 43(1): 50-6.

8. Santi D, Vezzani S, Granata AR, et al. Sperm quality and environment: A retrospective, cohort study in a Northern province of Italy. Environ Res. 2016; 150: 144-53.

9. Wu L, Jin L, Shi T, et al. Association between ambient particulate matter exposure and semen quality in Wuhan, China. Environ Int. 2017; 98: 219-28.

10. Lao XQ, Zhang Z, Lau AKH, et al. Exposure to ambient fine particulate matter and semen quality in Taiwan. Occup Environ Med. 2018; 75(2): 148-54.

11. Zhou N, Jiang C, Chen Q, et al. Exposures to Atmospheric PM10 and PM10-2.5 Affect Male Semen Quality: Results of MARHCS Study. Environ Sci Technol. 2018; 52(3): 1571-81.

12. Santi D, Magnani E, Michelangeli M, et al. Seasonal variation of semen parameters correlates with environmental temperature and air pollution: A big data analysis over 6 years. Environ Pollut. 2018; 235: 806-13.

13. Nobles CJ, Schisterman EF, Ha S, et al. Ambient air pollution and semen quality. Environ Res. 2018; 163: 228-36.

14. Huang X, Zhang B, Wu L, Zhou Y, Li Y, Mao X, et al. Association of Exposure to Ambient Fine Particulate Matter Constituents With Semen Quality Among Men Attending a Fertility Center in China. Environ Sci Technol. 2019 May 21;53(10):5957-5965.
